# Genetic Loci Associated with Plasma Phospholipid n-3 Fatty Acids: A Meta-Analysis of Genome-Wide Association Studies from the CHARGE Consortium

**DOI:** 10.1371/journal.pgen.1002193

**Published:** 2011-07-28

**Authors:** Rozenn N. Lemaitre, Toshiko Tanaka, Weihong Tang, Ani Manichaikul, Millennia Foy, Edmond K. Kabagambe, Jennifer A. Nettleton, Irena B. King, Lu-Chen Weng, Sayanti Bhattacharya, Stefania Bandinelli, Joshua C. Bis, Stephen S. Rich, David R. Jacobs, Antonio Cherubini, Barbara McKnight, Shuang Liang, Xiangjun Gu, Kenneth Rice, Cathy C. Laurie, Thomas Lumley, Brian L. Browning, Bruce M. Psaty, Yii-Der I. Chen, Yechiel Friedlander, Luc Djousse, Jason H. Y. Wu, David S. Siscovick, André G. Uitterlinden, Donna K. Arnett, Luigi Ferrucci, Myriam Fornage, Michael Y. Tsai, Dariush Mozaffarian, Lyn M. Steffen

**Affiliations:** 1 Cardiovascular Health Research Unit, Department of Medicine, University of Washington, Seattle, Washington, United States of America; 2 Clinical Research Branch, National Institute on Aging, National Institutes of Health, Baltimore, Maryland, United States of America; 3 Division of Epidemiology and Community Health, School of Public Health, University of Minnesota, Minneapolis, Minnesota, United States of America; 4 Center for Public Health Genomics, Division of Biostatistics and Epidemiology, University of Virginia, Charlottesville, Virginia, United States of America; 5 Institute of Molecular Medicine, University of Texas Health Science Center at Houston, Houston, Texas, United States of America; 6 Department of Epidemiology, University of Alabama at Birmingham, Birmingham, Alabama, United States of America; 7 Division of Epidemiology, Human Genetics, and Environmental Sciences, University of Texas Health Science Center at Houston, Houston, Texas, United States of America; 8 Department of Internal Medicine, University of New Mexico, Albuquerque, New Mexico, United States of America; 9 Duke Global Health Institute, Duke University, Durham, North Carolina, United States of America; 10 Geriatric Unit, Azienda Sanitaria Firenze (ASF), Florence, Italy; 11 Center for Public Health Genomics, University of Virginia, Charlottesville, Virginia, United States of America; 12 Institute of Gerontology and Geriatrics, Department of Clinical and Experimental Medicine, University of Perugia, Perugia, Italy; 13 Department of Biostatistics, School of Public Health, University of Washington, Seattle, Washington, United States of America; 14 Laboratory Medicine and Pathology, University of Minnesota, Minneapolis, Minnesota, United States of America; 15 Department of Statistics, University of Auckland, Auckland, New Zealand; 16 Department of Medicine, Division of Medical Genetics, University of Washington, Seattle, Washington, United States of America; 17 Departments of Epidemiology and Health Services, University of Washington, Seattle, Washington, United States of America; 18 Group Health Research Institute, Group Health Cooperative, Seattle, Washington, United States of America; 19 Medical Genetics Research Institute, Cedars-Sinai Medical Center, Los Angeles, California, United States of America; 20 Unit of Epidemiology, School of Public Health, Hebrew University-Hadassah, Jerusalem, Israel; 21 Division of Aging, Department of Medicine, Brigham and Women's Hospital, Harvard Medical School, Boston, Massachusetts, United States of America; 22 Boston VA Healthcare System, Boston, Massachusetts, United States of America; 23 Department of Epidemiology and Nutrition, Harvard School of Public Health, Boston, Massachusetts, United States of America; 24 School of Medicine and Pharmacology, University of Western Australia, Perth, Australia; 25 Department of Epidemiology, School of Public Health, University of Washington, Seattle, Washington, United States of America; 26 Department of Internal Medicine, Erasmus MC, Rotterdam, The Netherlands; 27 Division of Cardiovascular Medicine, Brigham and Women's Hospital, Harvard Medical School, Boston, Massachusetts, United States of America; University of Oxford, United Kingdom

## Abstract

Long-chain n-3 polyunsaturated fatty acids (PUFAs) can derive from diet or from α-linolenic acid (ALA) by elongation and desaturation. We investigated the association of common genetic variation with plasma phospholipid levels of the four major n-3 PUFAs by performing genome-wide association studies in five population-based cohorts comprising 8,866 subjects of European ancestry. Minor alleles of SNPs in *FADS1* and *FADS2* (desaturases) were associated with higher levels of ALA (*p* = 3×10^−64^) and lower levels of eicosapentaenoic acid (EPA, *p* = 5×10^−58^) and docosapentaenoic acid (DPA, *p* = 4×10^−154^). Minor alleles of SNPs in *ELOVL2* (elongase) were associated with higher EPA (*p* = 2×10^−12^) and DPA (*p* = 1×10^−43^) and lower docosahexaenoic acid (DHA, *p* = 1×10^−15^). In addition to genes in the n-3 pathway, we identified a novel association of DPA with several SNPs in *GCKR* (glucokinase regulator, *p* = 1×10^−8^). We observed a weaker association between ALA and EPA among carriers of the minor allele of a representative SNP in *FADS2* (rs1535), suggesting a lower rate of ALA-to-EPA conversion in these subjects. In samples of African, Chinese, and Hispanic ancestry, associations of n-3 PUFAs were similar with a representative SNP in *FADS1* but less consistent with a representative SNP in *ELOVL2*. Our findings show that common variation in n-3 metabolic pathway genes and in *GCKR* influences plasma phospholipid levels of n-3 PUFAs in populations of European ancestry and, for *FADS1*, in other ancestries.

## Author Summary

Circulating long-chain n-3 polyunsaturated fatty acids (PUFAs) derive from fatty fish or from the conversion of the plant n-3 PUFA by elongation and desaturation. We looked for common genetic markers throughout the genome that might influence plasma phospholipid levels of the four major n-3 PUFAs in five large studies and pooled the results. We found that levels of all four n-3 PUFAs were associated with genetic markers in known desaturation and elongation genes. We also found evidence that conversion of the plant n-3 PUFA to longer chain n-3 PUFAs is less effective in people with certain desaturation-gene markers, which could be important for people who do not eat fish. We also found a marker in a gene involved in glucose metabolism, called the glucokinase regulator, to be associated with one intermediate n-3 PUFA. Some of these findings were seen across multiple race/ethnicities. Overall, these results have implications for how genes and the environment interact to influence circulating levels of fatty acids.

## Introduction

High levels of n-3 polyunsaturated fatty acids (PUFA) in plasma phospholipids, cell membranes, and whole blood have been associated with lower risk of multiple diseases, including sudden cardiac death [1], fatal coronary heart disease [2], non-fatal myocardial infarction [3], heart failure [4], thickening of the carotid arteries [5], metabolic syndrome [6], breast cancer [7], chronic obstructive pulmonary disease [8], depression [9], [10] and dementia [11], [12].

**Figure 1:**
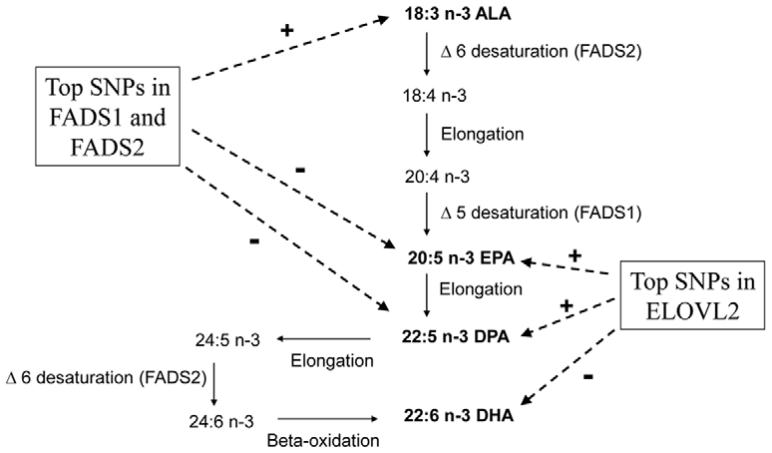
N-3 polyunsaturated fatty acid metabolic pathway and summary of genome-wide associations in pathway. The fatty acids indicated in bold were examined in this study. The genome-wide significant associations of two loci with each fatty acid are shown with dashed arrows. + and − signs indicate the direction of the associations.

N-3 PUFAs are derived directly from the diet, including the plant-derived essential fatty acid α-linolenic acid (ALA, 18:3n3) and the seafood-derived long-chain n-3 PUFAs eicosapentaenoic acid (EPA, 20:5n3) and docosahexaenoic acid (DHA, 22:6n3) [13], [14]. Long-chain n-3 PUFAs can also be produced from ALA by the series of desaturation and elongation steps in the pathway shown in Figure 1; docosapentaenoic acid (DPA, 22:5n-3) can be produced from EPA. The pathway enzymes may be a major source of circulating long-chain n-3 PUFAs in people who consume very little or no seafood. However, the conversion of ALA to EPA and DHA has been shown to be generally low [15]–[17], and it is not known whether common genetic variation in the pathway affects this conversion. The n-6 essential fatty acid linoleic acid (LA) is elongated to long-chain n-6 PUFAs by the same pathway enzymes and could also compete with the conversion of ALA to EPA; it is not known whether genetic variation affects such competition.

There is evidence of co-heritability of EPA and DHA levels in erythrocyte membrane phospholipids [18]. Investigation of genetic factors influencing PUFA levels has largely focused on candidate genes, such as the desaturase genes *FADS1* and *FADS2*, among participants of European ancestry [19]–[25]. Only one prior study reported a genome-wide association of n-3 PUFA levels evaluating total plasma n-3 PUFAs which includes triacylglycerols, phospholipids and free fatty acids, among 1075 participants [26]. The study found an association of EPA with variants in the FADS1 gene that reached genome-wide significance level; independent follow-up investigation showed associations of a selected *FADS1* variant with erythrocyte membrane levels of EPA, ALA and DPA and of an *ELOVL2* variant with DPA and DHA. These findings confirm an influence of *FADS1* and *ELOVL2* on selected n-3 PUFAs. However, statistical power may have been limited to confirm an influence of these genes on all four major n-3 PUFAs, of other genes in these pathways (e.g., *FADS2*), or of additional genes in other unknown biologic pathways. Prior studies have also not had adequate power to evaluate potential interaction between variation in these genes, EPA and DHA, and: (a) diet, (b) ALA levels, and (c) LA levels. In addition, there is limited information on genetic variation and n-3 PUFA levels in subjects of non-European ancestry.

To understand how common genetic variation affects n-3 PUFA phospholipid levels and potentially uncover novel associations, we conducted a meta-analysis of pre-planned genome-wide association analyses of plasma phospholipid n-3 PUFAs in 8,866 participants of European ancestry in five population-based studies, as part of the Cohorts for Heart and Aging Research in Genomic Epidemiology (CHARGE) Consortium [27]. We evaluated the four main n-3 PUFAs of the metabolic pathway, (ALA, EPA, DPA, and DHA) separately and accounted for the intercorrelations between these fatty acids. In addition, we investigated whether consumption of fatty fish or phospholipid levels of ALA and LA influenced the association of the identified genetic markers with EPA and DHA levels. Finally, we studied the most highly associated SNPs from the meta-analyses among samples of European ancestry in additional samples from African, Chinese and Hispanic ancestry.

## Results

### Meta-analysis of genome-wide associations of n-3 fatty acids

The study samples for the genome-wide association study (GWAS) comprised a total of 8,866 subjects of European ancestry. Table 1 shows sample size, demographic characteristics, fish consumption and phospholipid n-3 PUFA levels in the 5 cohorts that contributed to the study. Participants ranged from 21 to 102 years of age. Across the cohorts, mean levels of ALA varied from 0.14% to 0.44% of total fatty acids; EPA from 0.56% to 1.01%; DPA from 0.83% to 0.98%; and DHA from 2.29% to 5.09%. Relatively higher ALA levels were seen in the InCHIANTI cohort, likely reflecting differences in the composition of total plasma fatty acids (InCHIANTI) versus phospholipid fatty acids (ARIC, CARDIA, CHS, and MESA).[28]

**Table 1:** CHARGE cohorts descriptives.*

					Plasma phospholipid concentration, % of total fatty acids			
Cohort/Ancestry	N	Age	Women %	Fish intake¥	ALA^†^	EPA^†^	DPA^†^	DHA^†^
ARIC/European‡	3268	53.8 (5.6)	51.0	1.2 (0–24)	0.14 (0.05)	0.56 (0.30)	0.90 (0.17)	2.82 (0.88)
CHS/European‡	2326	72.0 (5.1)	61.3	2.0 (0–14)	0.15 (0.05)	0.59 (0.37)	0.83 (0.17)	2.97 (0.96)
CHS/African	427	72.9 (5.5)	68.3	NA	0.14 (0.05)	0.56 (0.29)	0.84 (0.17)	3.52 (0.93)
InCHIANTI/European‡	1075	68.4 (15.5)	54.9	1.6 (0–12)	0.44 (0.25)	0.61 (0.22)	NA	2.29 (0.77)
CARDIA/European‡	1507	45.8 (3.4)	53.3	1.0 (0–31)	0.19 (0.09)	0.85 (0.62)	0.94 (0.21)	3.09 (1.12)
CARDIA/African	493	44.6 (3.8)	60.7	1.2 (0–18)	0.17 (0.08)	0.67 (0.41)	0.92 (0.21)	3.27 (0.99)
MESA/European‡	690	61.6 (10.4)	53.3	0.6 (0–11)	0.18 (0.06)	0.88 (0.58)	0.95 (0.21)	3.66 (1.38)
MESA/African	631	61.6 (9.8)	54.2	0.6 (0–14)	0.16 (0.06)	0.89 (0.56)	0.98 (0.22)	4.57 (1.42)
MESA/Chinese	646	62.1 (10.4)	50.8	0.8 (0–11)	0.18 (0.07)	1.01 (0.65)	0.96 (0.24)	5.09 (1.46)
MESA/Hispanic	672	61.0 (10.0)	53.7	0.2 (0–7)	0.17 (0.06)	0.64 (0.41)	0.89 (0.19)	3.34 (1.23)

Descriptive characteristics of the cohorts of European ancestry included in the GWAS analyses and the cohorts of African, Chinese, and Hispanic ancestry included in the association analyses of selected SNPs.

*Values are mean (SD) unless specified otherwise.

‡Included in the meta-analyses of GWAS.

¥ Servings per week, median (range).

**Figure 2:**
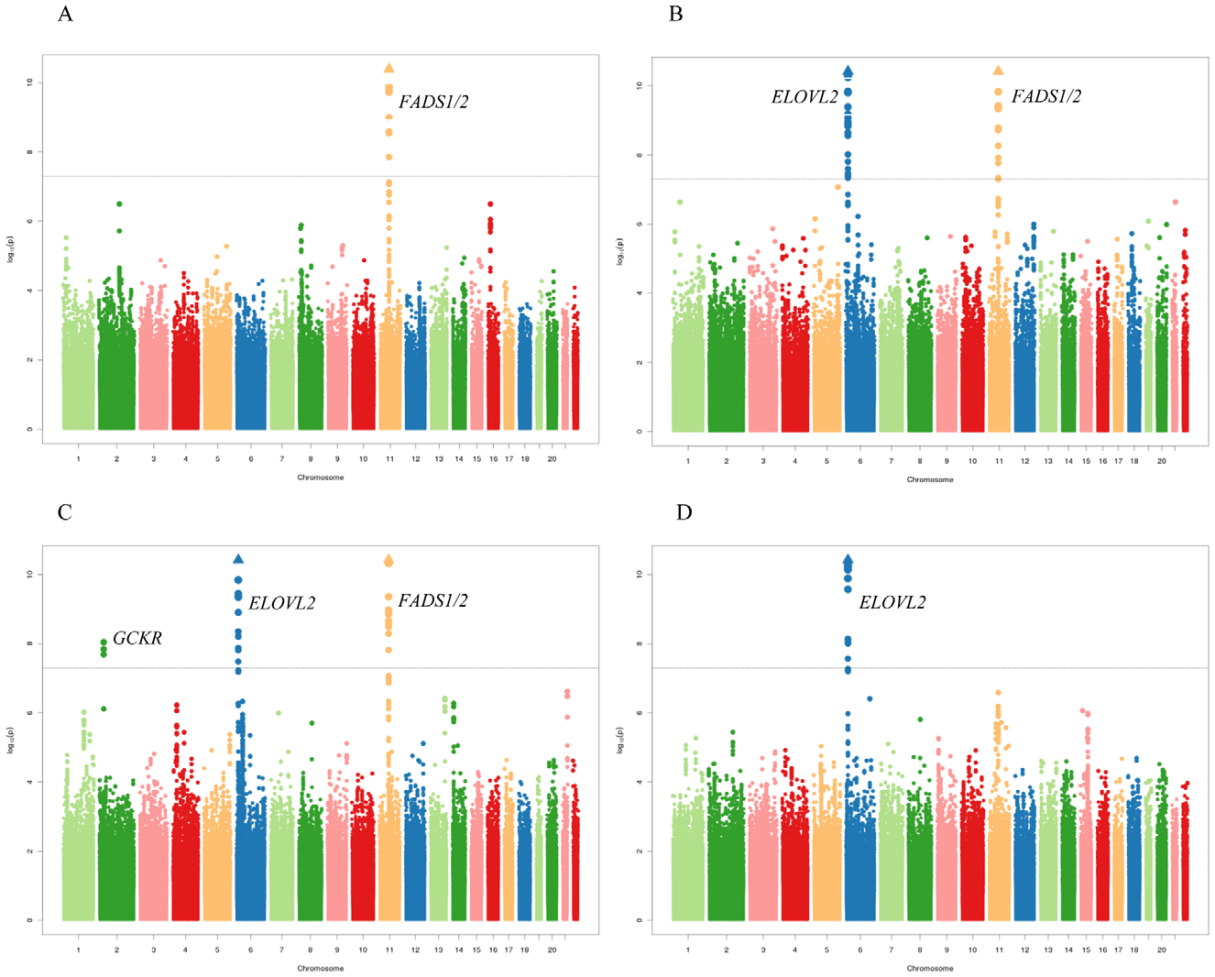
Meta-analysis of genome-wide associations with n-3 polyunsaturated fatty acids. A: α-linolenic acid (ALA), B: eicosapentaenoic acid (EPA), C: docosapentaenoic acid (DPA), D: docosahexaenoic acid (DHA). Associations were graphed by chromosome position and –log_10_ (p-value) up to p-values of 10^−10^. Triangles indicate additional SNPs with p-values less than 10^−10^. Genes of interest in the regions with significantly associated SNP variants are indicated.

**Figure 3:**
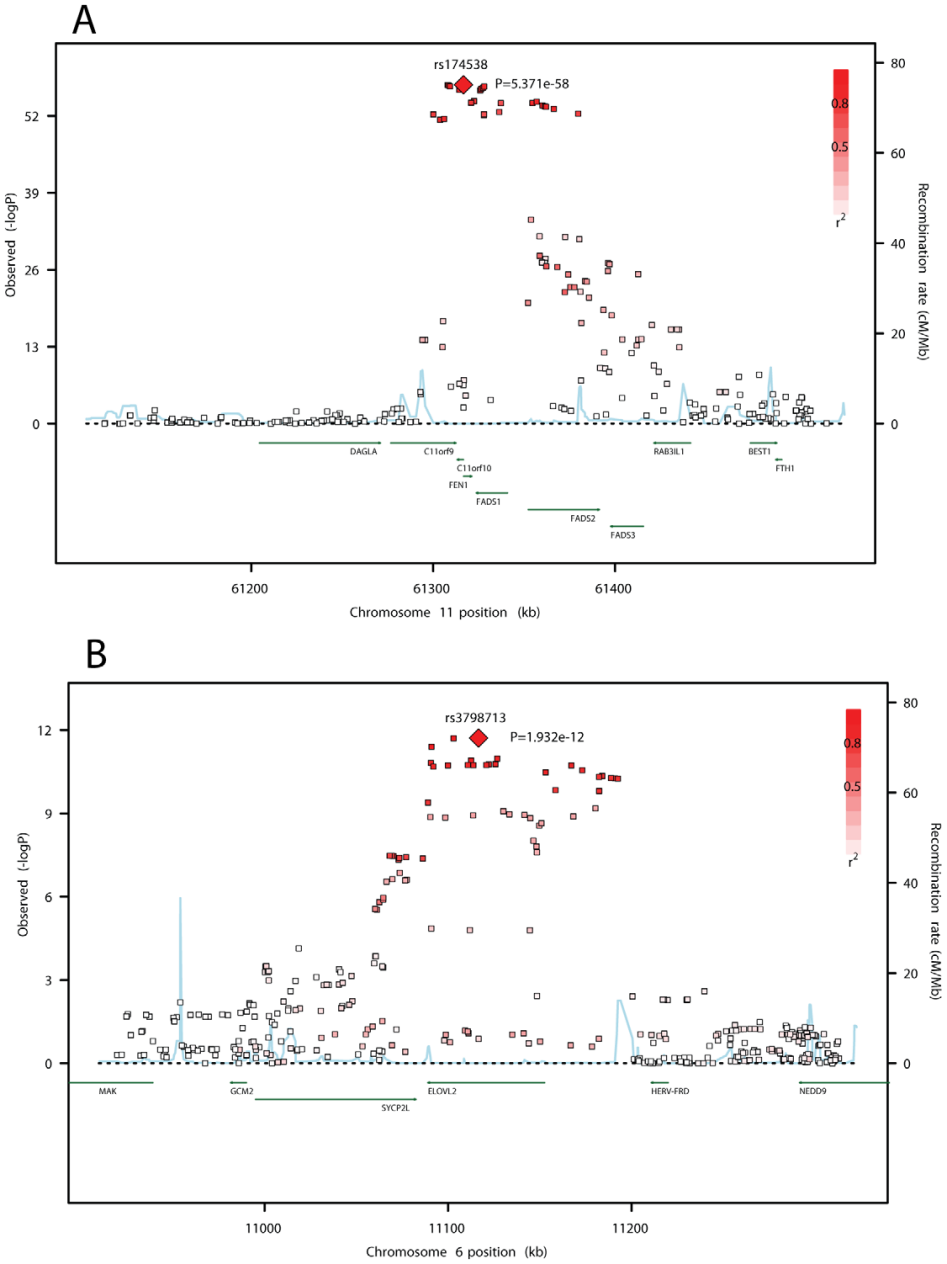
SNP association plots for EPA–associated regions. Genome-wide association significance level is plotted against the y-axis as –log_10_ (p-value). Genetic coordinates are as per NCI build 36. A) *FADS* cluster region. LD is indicated by color scale in relationship to marker rs174538. B) *ELOVL2* region. LD is indicated by color scale in relationship to marker rs3798713. The color scheme is red for strong linkage disequilibrium (LD; r^2^≥0.8) and fading color for lower LD.

Figure 2A–2D show the meta-analysis of the genome-wide association results for ALA, EPA, DPA, and DHA. Variation in one or both of two major genetic loci was associated with plasma phospholipid levels of each n-3 PUFA at genome-wide levels of significance. The two loci are illustrated in association plots for EPA (Figure 3). One locus, on chromosome 11q12.2, contained the *C11orf9/10*, *FEN1* and the desaturase genes *FADS1, FADS2* and *FADS3*. The other locus, on chromosome 6p24.2, contained *SYPC2L* and the elongase gene *ELOVL2*. Many highly correlated SNPs reached genome-wide significance in the associations with n-3 PUFAs (Table 2 and Table S1A–S1D). Variant alleles at SNPs in the chromosome 11 locus were associated with higher levels of ALA and lower levels of EPA and DPA, and variant alleles at SNPs in the chromosome 6 locus were associated with higher levels of EPA and DPA and lower levels of DHA (Table 2, Table 3, Table 4). From the meta-analysis results, we estimated that the most highly associated SNPs on chromosome 11 explained 3.8% of the variance of ALA, 2.0% of the variance of EPA and 8.6% of the variance of DPA. The most highly associated SNPs on chromosome 6 explained 0.4% of the variance of EPA, 2.8% of the variance in DPA and 0.7% of the variance in DHA.

**Table 2:** Loci associated with SNP markers with P-values <5.0×10^−8^.

			Most significant SNP			
Fatty acid (region)	Analysis adjustments	Number of SNPs|	SNP*, allele	MAF	P-value	Parameter Coefficient (CI)
ALA (11)	Main model†	75	rs174547,C/T	0.33	4×10^−64^	0.016 (0.014, 0.018)
ALA (11)	EPA‡	86	rs174547,C/T	0.33	7×10^−84^	0.019 (0.017, 0.021)
DPA (2)	Main model†	3	rs780094, T/C	0.41	9×10^−09^	0.017 (0.011, 0.022)
DPA (6)	Main model†	85	rs3734398, C/T	0.43	1×10^−43^	0.040 (0.035, 0.046)
DPA (6)	rs2236212, rs174547‡	22	rs12662634, A/G	0.18	3×10^−10^	−0.023 (−0.039, −0.021)
DPA (11)	Main model†	97	rs174547,C/T	0.33	4×10^−154^	−0.075 (−0.069, −0.080)
EPA(6)	Main model†	47	rs3798713, C/G	0.43	2×10^−12^	0.035 (0.025, 0.045)
EPA(6)	DHA‡	78	rs3798713, C/G	0.43	8×10^−35^	0.054 (0.045, 0.062)
EPA(6)	ALA‡	77	rs3798713, C/G	0.43	3×10^−12^	0.035 (0.025, 0.045)
EPA (11)	Main model†	78	rs174538, A/G	0.28	5×10^−58^	−0.083 (−0.094, −0.073)
EPA (11)	DHA‡	77	rs174535, C/T	0.31	2×10^−59^	−0.072 (−0.063, −0.080)
EPA (11)	ALA‡	87	rs174535, C/T	0.31	1×10^−84^	−0.097 (−0.088, −0.107)
DHA (6)	Main model†	57	rs2236212, C/G	0.43	1×10^−15^	−0.113 (−0.086, −0.141)
DHA (6)	EPA‡	76	rs2236212, C/G	0.43	7×10^−39^	−0.162 (−0.138, −0.186)

Results of the meta-analyses of n-3 fatty acid GWAS; main results with standard adjustments described in Methods, and additional results further adjusted for one other fatty acid or for selected SNPs.

†Adjustments of the cohort-specific GWAS; the main model included age, sex, and if appropriate study site and principal components to account for population structure.

‡Adjustment in addition to main model adjustments.

| Number of SNPs with p-values **<**5.0×10^−8^.

*The SNPs rs3798713, rs2236212 and rs3734398 are in perfect LD; rs174538, rs174535 and rs174547 are in LD with r^2^≥0.9. Positions for genome built 36.3 are 61327359 (rs174547), 27594741 (rs780094), 11090959 (rs3734398), 11182177 (rs12662634), 11116608 (rs3798713), 61316657 (rs174538), 61307932 (rs174535), 11103001 (rs2236212).

**Table 3:** Associations of rs174548 (G allele) with n-3 polyunsaturated fatty acids in samples of different ancestries.

			ALA		EPA		DPA		DHA	
	N	MAF	Beta* (SE)	p-value	Beta* (SE)	p-value	Beta* (SE)	p-value	Beta* (SE)	p-value
**European ancestry cohort** †										
ARIC	3268	0.29	0.013 (0.001)	7×10^−24^	−0.085 (0.008)	6×10^−27^	−0.075 (0.004)	1×10^−72^	−0.095 (0.024)	6×10^−05^
CARDIA	1507	0.30	0.019 (0.004)	3×10^−05^	−0.071 (0.030)	0.02	−0.069 (0.008)	4×10^−18^	−0.066 (0.046)	0.15
CHS	2326	0.28	0.019 (0.002)	2×10^−25^	−0.102 (0.011)	7×10^−20^	−0.078 (0.005)	2×10^−48^	−0.075 (0.029)	0.01
InCHIANTI	1075	0.24	0.033 (0.012)	0.005	−0.063 (0.009)	2×10^−11^	NA		−0.030 (0.037)	0.41
MESA	690	0.29	0.023 (0.003)	5×10^−11^	0.077 (0.027)	0.005	−0.056 (0.0012)	2×10^−06^	−0.117 (0.071)	0.10
Overall‡	8866	0.29	0.016 (0.001)	8×10^−59^	−0.081 (0.005)	8×10^−53^	−0.074 (0.003)	3×10^−138^	−0.076 (0.016)	1×10^−06^
**African ancestry cohort**										
CARDIA	1493	0.21	0.007 (0.004)	0.05	−0.070 (0.018)	9×10^−05^	−0.042 (0.009)	3×10^−06^	−0.122 (0.044)	0.005
CHS	426	0.20	0.009 (0.004)	0.03	0.011 (0.299)	0.71	−0.099 (0.015)	0.51	0.001 (0.088)	0.99
MESA	628	0.22	0.006 (0.004)	0.13	−0.028 (0.036)	0.44	−0.033 (0.014)	0.02	−0.018 (0.111)	0.87
Overall‡	2547	0.21	0.007 (0.002)	0.001	−0.061 (0.016)	1×10^−4^	−0.051 (0.007)	3×10^−14^	−0.089 (0.037)	0.02
**Chinese ancestry cohort**										
MESA	633	0.58	0.019 (0.004)	1×10^−06^	−0.046 (0.033)	0.17	−0.048 (0.012)	3×10^−05^	−0.008 (0.072)	0.91
**Hispanic ancestry cohort**										
MESA	661	0.52	0.016 (0.003)	3×10^−07^	−0.083 (0.020)	4×10^−05^	−0.078 (0.012)	3×10^−11^	−0.128 (0.053)	0.02

For each fatty acid, results are shown for the individual cohorts and for the meta-analyses among samples of European ancestry and samples of African ancestry.

*Beta-regression coefficient associated with one copy of the G allele.

†Included in the GWAS.

‡Meta-analysis of results of same ancestry cohorts.

**Table 4:** Associations of rs3734398 (C allele) with n-3 polyunsaturated fatty acids in samples of different ancestries.

			ALA		EPA		DPA		DHA	
	N	MAF	Beta* (SE)	p-value	Beta* (SE)	p-value	Beta* (SE)	p-value	Beta* (SE)	p-value
**European ancestry cohort** †										
ARIC	3268	0.42	0.0002 (0.001)	0.87	0.028 (0.008)	2×10^−04^	0.042 (0.004)	4×10^−24^	−0.119 (0.022)	8×10^−08^
CARDIA	1507		−0.002 (0.003)	0.52	0.055 (0.022)	0.01	0.037 (0.008)	1×10^−06^	−0.090 (0.041)	0.03
CHS	2326	0.43	0.003 (0.002)	0.08	0.044 (0.010)	7×10^−06^	0.040 (0.005)	1×10^−13^	−0.144 (0.027)	2×10^−07^
InCHIANTI	1075	0.43	−0.0007 (0.009)	0.94	0.037 (0.010)	2×10^−04^	NA		−0.092 (0.032)	0.004
MESA	690	0.42	0.0001 (0.003)	0.98	0.014 (0.029)	0.06	0.041 (0.011)	2×10^−04^	−0.065 (0.068)	0.34
Overall‡	8866	0.43	0.0008 (0.001)	0.37	0.035 (0.005)	4×10^−12^	0.040 (0.003)	1×10^−43^	−0.114 (0.014)	2×10^−15^
**African ancestry cohort**										
CARDIA	1493	0.25	−0.002 (0.004)	0.57	0.040 (0.017)	0.02	0.045 (0.008)	1×10^−07^	−0.077 (0.041)	0.06
CHS	426	0.25	0.003 (0.003)	0.37	−0.016 (0.022)	0.46	0.043 (0.013)	0.001	−0.191 (0.071)	0.008
MESA	628	0.25	−0.004 (0.004)	0.32	0.043 (0.035)	0.02	0.030 (0.013)	0.02	−0.042 (0.107)	0.69
Overall‡	2547	0.25	−0.0002 (0.002)	0.93	0.022 (0.013)	0.08	0.041 (0.006)	7×10^−12^	−0.099 (0.034)	0.003
**Chinese ancestry cohort**										
MESA	633	0.92	−0.005 (0.008)	0.51	−0.097 (0.071)	0.17	−0.005 (0.026)	0.85	−0.200 (0.154)	0.20
**Hispanic ancestry cohort**										
MESA	661	0.57	−0.002 (0.003)	0.56	−0.006 (0.021)	0.79	0.033 (0.012)	0.01	−0.110 (0.055)	0.05

For each fatty acid, results are shown for the individual cohorts and for the meta-analyses among samples of European ancestry and samples of African ancestry.

*Beta-regression coefficient associated with one copy of the G allele.

†Included in the GWAS.

‡Meta-analysis of results of same ancestry cohorts.

**Figure 4:**
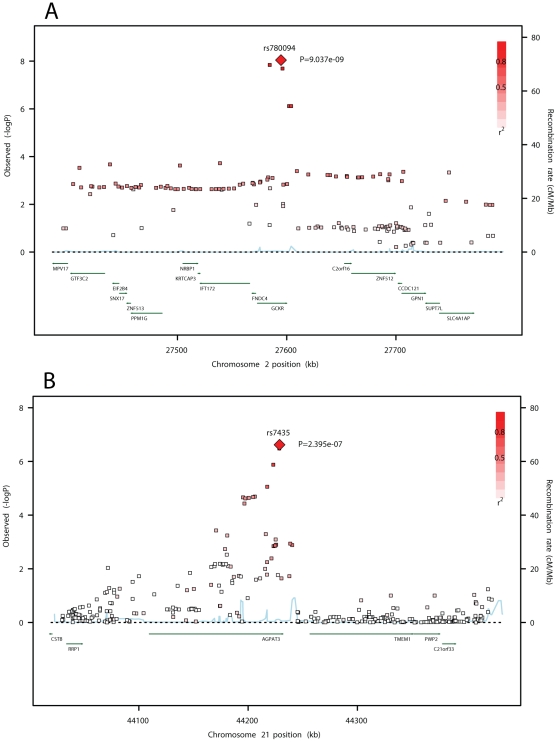
SNP association plots for DPA–associated regions. Genome-wide association significance level is plotted against the y-axis as –log_10_ (p-value). Genetic coordinates are as per NCBI build 36. A) *GCKR* region. LD is indicated by color scale in relationship to marker rs780094. B) *AGPAT3* region. LD is indicated by color scale in relationship to marker rs7435. The color scheme is red for strong linkage disequilibrium (LD; r^2^≥0.8) and fading color for lower LD.

Another genome-wide significant association with DPA was observed with SNPs on chromosome 2 in the *GCKR* gene (most associated SNP: rs780094, *p* = 9.0×10^−9^, Table 2 and Figure 4A). In addition, DPA showed a possible association with SNPs in *AGPAT3*, a gene on chromosome 21 involved in phospholipid metabolism (most associated SNP: rs7453, *p* = 2.4×10^−7^; Figure 4B and Table S1C).

Levels of EPA and DHA were correlated, as were levels of ALA and EPA. Spearman correlations in the 5 cohorts ranged from 0.26 to 0.57 for EPA-DHA and from 0.20 to 0.28 for ALA-EPA. When two outcomes are positively correlated but exhibit genetic associations in opposite directions, it is possible to increase the power of discovery efforts by adjusting the association between SNPs and one fatty acid trait for the other [29]. For this reason, we performed meta-analyses of genome-wide association results for ALA adjusted for EPA, EPA adjusted for ALA, EPA adjusted for DHA and DHA adjusted for EPA. These analyses did not reveal additional genome-wide significant loci, although the statistical significance of the adjusted associations was increased (Table 2). For example, the significance corresponding to the association of the SNP rs2236212 in *ELOVL2* with DHA increased from *p* = 1.3×10^−15^ to *p* = 7.0×10^−39^ with adjustment for EPA, with many more genome-wide significant associations in that region. In addition, the association of rs4985167 on chromosome 16 (*PDXDC1*) with ALA approached genome-wide significance with adjustment for EPA (*p* = 7.6×10^−8^).

To explore whether the signals in the chromosome 6 and 11 loci could be explained by one SNP alone, we performed GWAS of DPA with adjustment for rs2236212 and rs174547 in the ARIC, CARDIA, CHS and MESA cohorts and meta-analyzed the results. No new association was found in the chromosome 11 locus where the adjustment reduced the association for all other SNPs, from a minimum p-value of 1.1×10^−51^ to p>10^−6^ (not shown). In contrast, 22 SNPs in the *ELOVL2* region reached genome-wide significance, with ten of these SNPs previously undetected (Table 2, Table S1E). The A allele of rs12662634, the most highly associated SNP, had minor allele frequency of 0.18 and was associated with lower level of DPA (regression coefficient associated with one copy of A allele: −0.030, p = 2.7×10^−10^). This association was new, as the estimated effect in the unadjusted GWAS of DPA was 0.008 (p = 0.06). Among the 12 SNPs that had been detected before, rs9368564 showed the most significant association (regression coefficient and p-value associated with one copy of G allele: 0.027, p = 1.4×10^−9^, in the adjusted GWAS, and 0.048, p = 10^−40^, in the unadjusted GWAS). This result may reflect residual association in the adjusted analysis or yet another new association.

To explore if reducing the variability in EPA would reveal additional associations, we also performed GWAS of EPA adjusted for estimated fish intake. No additional associations beyond those previously seen for SNP in *FADS1/2* and *ELOVL2* were observed in these analyses (not shown). Finally, adjustment of the GWAS of the four n-3 PUFAs for levels of triglycerides, high density lipoprotein and low density lipoprotein did not materially change the results (not shown). For example, in the GWAS of DPA, the estimated effect of one copy of the T allele of rs780094 (*GCKR*) went from 0.0157 (p = 1.15×10^−8^) without adjustment to 0.0175 (p = 2.52×10^−9^) with adjustment, in meta-analyses that included 7663 participants.

### Association of top SNPs with n-3 PUFAs in samples from participants of African, Chinese, and Hispanic ancestry

To determine whether the gene-fatty acid associations were consistent across different ethnicities, we examined the associations of genotype at two representative SNPs with phospholipid n-3 PUFA levels, in samples of African, Chinese and Hispanic ancestry. Results of these analyses for the selected SNPs are shown in Table 3 and Table 4, together with meta-analysis and cohort-specific results among the samples of European ancestry. Frequency of the G allele of rs174548 (*FADS1*) was 0.29, 0.21, 0.58, 0.52 and frequency of the C allele of rs3734398 (*ELOVL2*) was 0.43, 0.25, 0.92, 0.57 in samples of European, African, Chinese and Hispanic ancestry respectively. Associations of rs174548 with n-3 PUFA were generally similar across all ancestries, with the G allele associated with higher ALA and lower long-chain n-3 PUFA levels, although associations did not always reach statistical significance, perhaps due to limited sample sizes (Table 3). The associations of rs3734398 with EPA, DPA and DHA were similar for samples of African ancestry versus European ancestry (Table 4). Among samples of Chinese ancestry, SNP rs3734398 was not highly polymorphic (C allele frequency of 92%) and no significant associations were detected. In samples of Hispanic ancestry, the C allele of rs3734398 was associated with higher DPA and lower DHA, but it was not associated with EPA.

### Interactions

**Figure 5:**
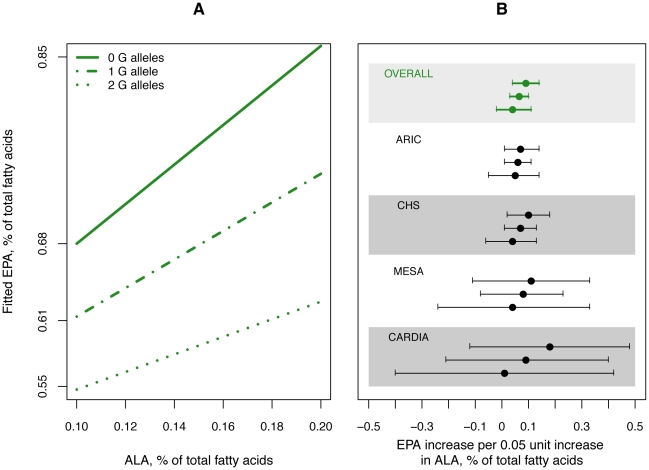
Influence of variation in rs1535 on the estimated association of plasma phospholipid ALA levels with EPA levels. Panel A illustrates the association of ALA with EPA in carriers of 0, 1, and 2 copies of rs1535 G allele with fitted lines constructed from meta-analyzed estimates. Panel B shows the % change in EPA corresponding to a 0.05 unit increase in ALA in carriers of 0 (top estimate), 1 (middle estimate), 2 (bottom estimate) copies of rs1535 G allele.

We evaluated several potential interactions in the samples of European ancestry, with statistical significance defined at alpha = 0.004 (0.05 divided by 13 tested interactions). We found little evidence that fatty fish consumption (≥*vs.* <0.6 servings/week) modified the associations of rs1535 (*FADS2*) or rs3734398 (*ELOVL2*) with levels of DHA or EPA. We also did not observe any interaction between plasma phospholipid levels of LA (continuous linear) and genotype at these two SNPs on the levels of DHA or EPA. Plasma phospholipid levels of ALA (continuous linear) also did not modify the association of genotype at these two SNPs with levels of DHA, or of genotype at rs3734398 with levels of EPA. However, there was a significant interaction of ALA with rs1535 genotype and EPA levels (meta-analyzed interaction coefficient *p* = 9.3×10^−7^), illustrated in Figure 5. Per one SD unit (0.05% of total fatty acids) increase in ALA, EPA levels increased by 0.086% of total fatty acids (23% of one SD) in the absence of the minor allele (G); by 0.063% (17% of one SD) in the presence of one copy (G-); and by 0.036% (10% of one SD) in the presence of two copies (GG).

## Discussion

We report here the results of the largest GWAS of plasma phospholipid n-3 PUFAs to date, with 8,866 participants of European ancestry. The associations of the two top hits on chromosomes 6 and 11 are shown in context of the n-3 PUFA pathway in Figure 1. Genetic variation in the desaturase genes *FADS1* and *FADS2* was associated with higher levels of ALA and lower levels of EPA and DPA suggesting genetic variants that affect the conversion of ALA to EPA and DPA. In the main analyses, genetic variation in the elongase gene *ELOVL2* was associated with higher levels of EPA and DPA and lower levels of DHA, suggesting variants that decrease the conversion of EPA and DPA to DHA. These reciprocal associations support a role of genetic variation in the pathway for circulating levels of n-3 PUFAs in free-living populations.

The associations of *FADS1/2* and *ELOVL2* with n-3 PUFAs were generally consistent with the previous GWAS in InCHIANTI (total plasma n-3 PUFAs) and follow-up candidate replication in the Genetics of Lipid-Lowering Drugs and Diet Network (GOLDN) Study (erythrocyte n-3 PUFAs) [26]. In that prior study, only the association of variation in *FADS1* with EPA reached genome-wide significance. Follow-up investigations in GOLDN suggested associations of *FADS1* gene variation with ALA and DHA, and of *ELOVL2* gene with DHA and DPA, such as the ones reported here [26]. In the present larger meta-analyses, these prior suggestive associations reached genome-wide significance, providing a definite picture of the association of genes in the PUFA pathway with phospholipid n-3 PUFAs. While the InCHIANTI cohort showed similar estimates of association with ALA, EPA and DHA as the other cohorts in the present study, further studies are needed to further explore genetic associations of n-3 PUFAs in triglycerides and other fatty acid compartments.

Using ratios of n-6 PUFAs as proxy of desaturase activities, Bokor et al reported that the minor allele of *FADS1* rs174546 was associated with lower delta-5 desaturase activity and the minor allele of *FADS2* rs968567 was associated with higher delta-6 desaturase activity [30]. We found both rs174546 and rs968567 were associated with higher levels of ALA and lower levels of EPA and DPA at genome-wide significance level (Table S1A–S1C). Furthermore, adjustment of the GWAS of DPA for the most associated SNP from the *FADS1/2* genes and the most associated SNP from *ELOVL2* did not reveal additional associations on chromosome 11. This result suggests that each associated SNP conveyed the information contained in the other SNPs of the same broad region on chromosome 11. In contrast, we found evidence for two independent associations, exemplified by rs2236212 and rs12662634, in the region of the *ELOVL2* gene.

The associations of the *FADS1/2* and *ELOVL2* genes with the phospholipid levels of EPA and DHA did not vary depending on the frequency of fatty fish consumption, suggesting the genetic effects are independent of fish intake at the levels of consumption in the studied populations. The absence of interaction of *FADS1/2* by fish consumption is consistent with findings from the Netherlands KOALA Birth Cohort Study [31], in which higher levels of fish consumption were associated with similar slopes of plasma phospholipid EPA and DHA levels among individuals with 0, 1, or 2 copies of minor *FADS1/2* alleles. The associations of *FADS1/2* and *ELOVL2* genes with EPA and DHA also did not vary depending on phospholipid LA. Linoleic acid is desaturated and elongated to n-6 metabolites (e.g. arachidonic acid) using the same desaturases and elongase(s) as ALA, and in dietary trials, higher dietary LA reduces plasma phospholipid EPA [32]. Animal experiments suggest that high dietary LA inhibits *FADS2* gene expression [33]. Our findings do not support an influence of LA on the association of genetic variation in the pathway with n-3 PUFA levels, at levels of LA consumption in these cohorts.

We report for the first time a GWAS of DPA, a central intermediate in the n-3 fatty acid pathway (Figure 1). While present in small quantities in fatty fish, DPA plasma levels appear unrelated to dietary intake [3], suggesting a primarily metabolic origin. Supporting this, we found that DPA exhibited stronger genetic associations than the other n-3 PUFAs.

In addition to its association with variants of desaturase and elongase genes, DPA was associated with variation in the glucokinase regulator gene *GCKR,* a pleiotropic gene associated with multiple outcomes in GWAS [34]. The T allele of rs780094 is associated with lower fasting glucose and insulin [35] and with higher triglycerides [36]–[38]; this allele was associated with higher DPA levels in the present study, and the association was independent of triglyceride levels. Given the known influence of long-chain n-3 PUFAs on hepatic triglyceride production [39] and possibly glucose-insulin homeostasis [40], the mechanism of potential pleiotropic effects of this allele on both DPA and these pathways merit further attention.

We found a potential association of DPA with *AGPAT3,* a gene encoding 1-acylglycerol-3-phosphate O-acyltransferase 3. DPA is a known substrate for the *AGPAT3* protein, which transfers a fatty acid in sn-2 position of lysophosphatic acid, a step in the phospholipid biosynthesis pathway. A possible association of DPA with *AGPAT3* variation supports an origin of phospholipid DPA from *de novo* phospholipid synthesis. In contrast, phospholipid EPA and DHA often originate to a greater extent from diet and are predominantly integrated into phospholipids by the process of acyl-chain remodelling [41]. The genetic associations reported here together with growing evidence of the association of DPA with lower risk of coronary heart disease [3], [42], [43] should stimulate further work on factors regulating this fatty acid.

The GWAS of ALA adjusted with EPA revealed a possible new association of phospholipid ALA with variation in *PDXDC1*. The *PDXDC1* protein, a vitamin B6-dependent decarboxylase, is expressed preferentially in the intestine [44], but its function is not known. Animal studies support an influence of dietary vitamin B6 (pyridoxal) on serum and liver levels of ALA and other PUFAs [45], [46], which has been interpreted as an effect on desaturase enzymes activity. The association of *PDXDC1* with ALA, if confirmed in other studies, raises the possibility of another vitamin B6-dependent protein affecting ALA, for example through involvement in intestinal ALA absorption.

In addition to an overall association of *FADS1/2* variation with less ALA and more EPA, we found that the minor G allele of rs1535 was associated with a reduction of the magnitude of the association between ALA and EPA. In persons with two copies of the G allele, the association of ALA with EPA was less than half the association observed in persons with two copies of the A allele. These results suggest an influence of variation in *FADS1/2* on the rate of conversion of ALA into EPA. This conversion is of great clinical and public health interest, given the evidence for importance of long-chain n-3 PUFAs (such as EPA) in many chronic diseases, their limited dietary supply worldwide, and the much greater potential supply of plant-derived ALA. On average, the conversion of ALA to EPA is quite low [47]. Prior tracer studies in humans have shown that the majority of dietary ALA is beta-oxidized for energy or directed into long-term storage as triglycerides, with less than 5% being incorporated into phospholipids where ALA is more readily converted to EPA [16], [17], [47]. Genetic variation that increases or decreases the rate of conversion of ALA to EPA could have implications for individual-based recommendations for consumption of plant- versus seafood-derived n-3 PUFA. The genetic variation may also indicate novel targets for drugs that may increase this conversion.

The associations of a representative SNP in the *FADS1/2* genes observed in the meta-analyses of samples of European ancestry were generally similar in samples of African, Chinese, and Hispanic ancestry. Associations of *ELOVL2* were less consistent in different ancestries. However, the frequency of the *ELOVL2* rs3734398 G allele varied substantially with ancestry, from 25% in African samples to 92% in Chinese samples. Lack of association may be due to inadequate statistical power, chance, different background diet [14], or possible race/ethnic differences in the activity of elongases from the *ELOVL2* and *ELOVL5* genes.

Our study, the largest GWAS to-date of fatty acid biomarkers, demonstrates key associations of genetic variation with phospholipid n-3 PUFA levels, including genes in the n-3 PUFA metabolic pathway and, for DPA, novel pathways including the pleiotropic gene *GCKR.* Our results also imply that common variation may result in less efficient conversion of ALA to EPA.

## Materials and Methods

### Ethics statement

All cohort participants gave written informed consent, including consent to participate in genetic studies. All studies received approval from local ethical oversight committees.

### Study samples

The data were obtained from 2 cohort studies in the CHARGE Consortium, the Atherosclerosis Risk in Communities (ARIC) Study and the Cardiovascular Health Study (CHS), and 3 additional cohort studies, the Coronary Artery Risk Development in Young Adults (CARDIA) Study, the Invecchiare in Chianti (InCHIANTI) Study, and the Multi-Ethnic Study of Atherosclerosis (MESA).

### Fatty acid measurements

In all cohorts but InCHIANTI, plasma phospholipids were first isolated by thin layer chromatography; fatty acids were then separated by gas chromatography. In InCHIANTI, total plasma fatty acids were measured using a similar gas chromatography technique. Details of fatty acid measurements are provided in Text S1. Levels of EPA, DHA, ALA and DPA were expressed as % of total fatty acids.

### Imputation and statistical analysis

Genotyping was done in each cohort separately using high-density SNP marker platforms (ARIC, CARDIA and MESA - Affymetrix 6.0, CHS - Illumina 370, InCHIANTI - Illumina 550). Samples with call rates below 95% (ARIC, CARDIA, MESA), or 97% (CHS, InCHIANTI) at genotyped markers were excluded. Genotypes were imputed to approximately 2.5 million HapMap SNPs by using either MACH [48] (ARIC, InCHIANTI), BIMBAM [49] (CHS), BEAGLE [50] (CARDIA) or IMPUTE [51] (MESA). SNPs for which testing Hardy Weinberg equilibrium resulted in *p<*10^−5^ (CHS) or *p*<10^−6^ (ARIC) were excluded from imputation. SNPs with minor allele frequency (MAF) ≤1% were excluded from the meta-analyses. Additional details on genotyping and imputation per cohort are provided in Text S1.

Association analysis between genotype and each fatty acid was done separately within each study cohort according to a pre-specified plan. All studies conducted linear regression analysis using an additive genetic model, i.e. regression of phenotype on the number of reference alleles, or equivalently the imputed dosage for imputed genotypes. All analyses were adjusted for age, sex, and site of recruitment where appropriate, and used robust standard errors. In addition, CARDIA, CHS and MESA analyses were adjusted for principal components to account for possible population genetic substructure. The results in InCHIANTI included in the present study have been previously published [26].

### Meta-analysis of main effects

For each SNP and fatty acid, GWAS-specific results were combined using inverse-variance weighted meta-analysis in METAL (www.sph.umich.edu/csg/abecasis/metal). Genomic control correction was applied to each study prior to the meta-analysis. Genomic control correction factors ranged from 1.00 to 1.07 (ALA), 1.00–1.08 (EPA), 1.00–1.03 (DPA) and 1.01–1.13 (DHA). P-values less than 5×10^−8^ were considered significant. Because total plasma levels of ALA (measured in InCHIANTI) are higher than plasma phospholipid levels of ALA (measured in the other cohorts), we performed a z-score based meta-analysis of ALA with the 5 cohorts as a sensitivity analysis. Results did not differ from that of inverse-variance weighted meta-analysis, i.e. from those presented. The proportion of fatty acid variance explained by a particular variant allele was calculated from the formula.

(β^2^*2*MAF(1-MAF))/Var(Y), where β is the regression coefficient for one copy of the allele, MAF is the minor allele frequency and Var(Y) is the variance of the fatty acid.

### Interaction analyses

We tested 13 interactions using cross-products in the linear regression models. Two of the most associated SNPs available in all cohorts (rs1535 in *FADS2* and rs3734398 in *ELOVL2*) were chosen for investigation of interactions with a) fatty fish intake (dichotomized at 0.6 servings/week, a cut-point around the 25^th^ percentile of fish consumption in the CHS and ARIC cohorts), b) plasma phospholipid ALA levels (continuous linear) and c) plasma phospholipid LA levels (continuous linear) on the outcomes of EPA and DHA. Additionally, we tested the interaction between rs1535 and plasma phospholipid EPA with DHA levels as the outcome. Interaction coefficients from cohort-specific analyses were meta-analyzed. For interactions of SNPs with ALA on the outcomes of EPA and DHA, we performed z-score meta-analysis with all the cohorts to assess statistical significance, and inverse-variance weighted meta-analysis excluding InCHIANTI to estimate the magnitude of the interaction. P-values less than 0.004 (0.05/13 tests) were considered significant for the interactions.

### Analyses of selected SNPs in cohorts of African, Chinese, and Hispanic ancestry

We investigated the association of two selected SNPs which had been directly genotyped as part of candidate gene studies in the African American cohort in CARDIA and the African, Chinese and Hispanic American cohort in MESA, and which were available from genome-wide scans on African Americans in the CHS cohort. We used linear regression and additive models as described above. Results in the 3 African American cohorts were meta-analyzed using inverse-variance weighted meta-analysis.

## Supporting Information

Text S1 Details of participating cohorts.
(DOC)

Table S1 Supplementary results from the CHARGE consortium. A. Comprehensive results for ALA with *p*<5*10^−6^. B. Comprehensive results for EPA with *p*<5*10^−6^. C. Comprehensive results for DPA with *p*<5*10^−6^. D. Comprehensive results for DHA with *p*<5*10^−6^. E. Results for DPA adjusted for rs2236212 and rs174547 with *p*<5*10^−8^.
(DOC)
