# Supplementary material for: Genetic Loci Associated with Plasma Phospholipid n-3 Fatty Acids: A Meta-Analysis of Genome-Wide Association Studies from the CHARGE Consortium
Source: PLoS Genet. 2011 Jul 28;7(7):e1002193. doi: 10.1371/journal.pgen.1002193 (PMC3145614; doi:10.1371/journal.pgen.1002193)
Supplement: Text S1 — Details of participating cohorts. (DOC) [file pgen.1002193.s001.doc]

**Text S1: Details of Participating Cohorts**

**Study Samples**

Participants for the current analysis were drawn from 5 cohort studies, including the Atherosclerosis Risk in Communities Study (ARIC), the Cardiovascular Health Study (CHS), the Coronary Artery Risk Development in Young Adults Study (CARDIA), the Invecchiare in Chianti Study (InCHIANTI), and the Multi-Ethnic Study of Atherosclerosis (MESA). These groups comprise the CHARGE (Cohorts for Heart and Aging Research in Genome Epidemiology) Consortium. All participants provided informed consent. Local ethical committees at each institution approved the individual study protocols.

**Study Samples, Phenotype, and Genotyping in the Participating Cohorts**

**The Atherosclerosis Risk in Communities Study**

The ARIC study is a multi-center prospective investigation of atherosclerotic disease in a predominantly bi-racial population [1]. White and African American men and women aged 45-64 years at baseline were recruited from 4 communities: Forsyth County, North Carolina; Jackson, Mississippi; suburban areas of Minneapolis, Minnesota; and Washington County, Maryland. A total of 15,792 individuals participated in the baseline examination in 1987-1989, with follow-up examinations in approximate 3-year intervals, during 1990-1992, 1993-1995, and 1996-1998.

ARIC Study samples were genotyped using the Affymetrix Genome-Wide Human SNP Array 6.0 (Santa Clara, California); for the current analysis only white participants were analyzed. Sample exclusion criteria included discordant with previous genotype data (n=83), genotypic and phenotypic sex mismatch (n=32), suspected first-degree relative of an included individual based on genotype data (n=297), genetic outlier as assessed by Identity by State (IBS) using PLINK [2] and >8 SD along any of the first 10 principal components in EIGENSTRAT [3] with 5 iterations (n=322). Autosomal SNPs were used for imputation after exclusion of SNPs with HWE deviation p<5 x 10‾^5^, call rate <95%, or MAF<1%.

Fatty acids were measured in EDTA plasma that had been frozen at -70˚C. Fatty acid assays were performed at the Collaborative Studies Clinical Laboratory at Fairview-University Medical Center (Minneapolis, MN) as previously described [4]. Lipids were extracted with chloroform/methanol and separated by thin layer chromatography. Fatty acid methyl esters were prepared from the phospholipid fraction and separated by gas chromatography using an HP-5890 gas chromatograph (Hewlett- Packard, Palo Alto, CA) with a 100-m capillary Varian CP7420 column. We identified 29 fatty acids. The concentration of each fatty acid was expressed as to percentage of total fatty acids.

**The Cardiovascular Health Study**

The CHS is a population-based longitudinal study of risk factors for cardiovascular disease and stroke in adults 65 years of age or older, recruited at four field centers (Forsyth County, NC; Sacramento County, CA; Washington County, MD; Pittsburgh, PA) [5]. Overall, 5201 predominantly Caucasian individuals were recruited in 1989-1990 from random samples of Medicare eligibility lists, followed by an additional 687 African-Americans recruited in 1992-1993 (total n=5,888). The CHS genome-wide association study (GWAS), which had the primary aim of studying incident cardiovascular events, focused on 3980 CHS participants who were free of clinical cardiovascular disease at study baseline, consented to genetic testing, and had DNA available for genotyping. A total of 1,908 persons were excluded from the GWAS study sample due to the presence at study baseline of coronary heart disease, congestive heart failure, peripheral vascular disease, valvular heart disease, stroke, or transient ischemic attack. Fatty acids were measured on samples collected in the 3^rd^ year of followup.

CHS Study samples were genotyped using the Illumina HumanCNV370-Duo BeadChip system. Because the other cohorts were predominantly of European descent, the African American participants were excluded from the genome-wide association analysis. Genotyping was successful in 3,291 Caucasian subjects. Participants were eligible for the present investigation if their genotyping was complete and they had available phenotype information. Samples with call rate <95% were excluded. A total of 306,655 autosomal SNPs were used in imputation after filtering out SNPs with HWE deviation p≤1 x 10‾^5^, call frequency ≤97%, zero heterozygote frequency, missing from dbSNP, and >1 duplicate or Mendelian inconsistency.

Blood was drawn after a 12-hour fast and stored at -70^o^C. Measurements were performed at the Fred Hutchinson Cancer Research Center, providing quantitative measurement of 42 fatty acids. Total lipids were extracted from plasma using methods of Folch, and phospholipids separated from neutral lipids by one-dimensional TLC. Fatty-acid-methyl-ester (FAME) samples were prepared by direct transesterification using methods of Lepage and separated using gas chromatography1 (Agilent5890 gas-chromatograph-FID-detector; Supelco fused-silica 100m capillary column SP-2560; initial 160°C 16 min, ramp 3.0°C/min to 240°C, hold 15 min). Identification, precision, and accuracy were continuously evaluated using model mixtures of known FAMEs and established in-house controls, with identification confirmed by GC-MS at USDA (Peoria, IL). CVs were <3% for most fatty acids.

**The Cardiovascular Risk Factors in Young Adults Study**

The CARDIA Study is a prospective multicenter study with 5115 adults Caucasian and African American participants of the age group 18-30 years, recruited from four centers. The recruitment was done from the total community in Birmingham, AL, from selected census tracts in Chicago, IL and Minneapolis, MN; and from the Kaiser Permanente health plan membership in Oakland, CA. The details of the study design for the CARDIA study have been published before [6]. Seven examinations have been completed since initiation of the study in 1985–1986, respectively in the years 0, 2, 5, 7, 10, 15 and 20. Written informed consent was obtained from participants at each examination and all study protocols were approved by the institutional review boards of the participating institutions.

CARDIA Study samples from were genotyped using the Affymetrix Genome-Wide Human SNP Array 6.0 (Santa Clara, California); only participants of European descent were included in the GWAS analyses. Genotyping was completed for 1720 individuals with a sample call rate ≥ 98%. A total of 578,568 SNPs passed quality control (MAF ≥ 2%, call rate ≥ 95%, HWE ≥ 10^-4^) and were used for imputation. For this study, complete genotype and phenotype information were available for 1507 individuals.

We also genotyped selected SNPs for participants of African descent using the TaqMan assay (Applied Biosystems, Foster City, CA) as previously described [[18]](http://www.sciencedirect.com.ezproxyhost.library.tmc.edu/science?_ob=ArticleURL&_udi=B6WN4-4Y29T4M-C&_user=5674961&_coverDate=08%2F31%2F2010&_rdoc=1&_fmt=high&_orig=search&_sort=d&_docanchor=&view=c&_acct=C000003838&_version=1&_urlVersion=0&_userid=5674961&md5=135e3bb97b818c043cde378ba2b623dc#bbib18) M. Fornage and P.A. Doris, Single-nucleotide polymorphism genotyping for association studies. In: J.P. Fennell and A.H. Baker, Editors, *Hypertension methods and protocols*, Humana Press, Totowa (NJ) (2004), pp. 159–172.[7]. Primer and probes are available from the authors upon request. Polymorphism genotyping in the CARDIA study adheres to a rigorous quality control program, which includes barcode identification of samples, robotic sample handling, and blind replicate genotype assessment on 5% of the total sample (n = 219). The overall genotyping rate with the TapMan assay was 97%, and the concordance rate for blind duplicates was greater than 99%.

Fatty acids were measured in EDTA plasma, frozen at –70°C, using methods previously described by Cao et al [4]. Lipids are extracted from the plasma using a chloroform/methanol extraction method and the cholesterol esters, triglyceride, phospholipids and free fatty acids are separated by thin layer chromatography. The fatty acid methyl esters are obtained from the phospholipids and are detected by gas chromatography flame ionization. Individual fatty acids are expressed as a percent of total fatty acids. 28 fatty acids were identified.

**The Invecchiare in Chianti Study**

The InCHIANTI study is a population-based epidemiological study performed in a sample of the population living in the Chianti region of Tuscany, Italy. 1616 residents were selected from the population registry of Greve in Chianti and Bagno a Ripoli. The participation rate was 90% (n=1453), and the subjects age ranged between 21 and 102 years. Overnight fasted blood samples were used for genomic DNA extraction, and measurement of fatty acids. For this studyy, we used data from 1206 subjects with complete phenotype and genotype data.

InCHIANTI Study samples were genotyped using the Illumina *fill in Chip* system. Genotyping was completed for 1210 subjects with a sample call rate >97%, heterozygosity rates > 0.3 and correct sex specification. A total of 495,343 autosomal SNPs that passed quality control (MAF>1%, completeness >99%, HWE > 10^-4^) were used for imputation.

Fatty acids were measured on aliquots of plasma that had been continuously stored at -80 C as described previously[8]. Fatty acid methyl esters (FAME) were prepared through transesterification using Lepage and Roy’s method [9] with modification Rodriguez-Palmero et al [10]. Separation of FAME was carried out on an HP-6890 gas chromatograph (Hewlett- Packard, Palo Alto, CA) with a 30-m fused silica column (HP-225; Hewlett-Packard). FAMEs were identified by comparison with pure standards (NU Chek Prep, Inc., Elysian, MA). We identified 20 fatty acids. For quantitative analysis of fatty acids as methyl esters, calibration curves for FAME (ranging from C14:0 to C24:1) were prepared by adding six increasing amounts of individual FAME standards to the same amount of internal standard (C17:0; 50xg). The correlation coefficients for the calibration curves of fatty acids were in all cases higher than 0.998 in the range of concentrations studied. Fatty acid concentrations were expressed as a percentage of total fatty acids. The coefficient of variation for all fatty acids was on average 1.6% for intraassay and 3.3% for interassay.

**The Multi-Ethnic Study of Atherosclerosis**

The MESA Study is a study of the characteristics of subclinical cardiovascular disease (disease detected non-invasively before it has produced clinical signs and symptoms) and the risk factors that predict progression to clinically overt cardiovascular disease or progression of the subclinical disease.[11] MESA researchers study a diverse, population-based sample of 6,814 asymptomatic men and women aged 45-84. Thirty-eight percent of the recruited participants are white, 28 percent African-American, 22 percent Hispanic, and 12 percent Asian, predominantly of Chinese descent, as well as 2,128 additional individuals from 594 families recruited through MESA Family by utilizing the existing MESA framework, yielding 3,026 sibpairs divided between African Americans and Hispanic-Americans. Participants were recruited from six field centers across the United States: Wake Forest University, Columbia University, Johns Hopkins University, University of Minnesota, Northwestern University and University of California - Los Angeles.

MESA and MESA Family samples were genotyped using the Affymetrix Genome-Wide Human SNP Array 6.0 (Santa Clara, California); for the current meta-analysis only self-reported Caucasian participants were analyzed, while MESA Chinese, African American and Hispanic samples are included in the look-up of top SNPs. Sample exclusion criteria included heterozygosity > 53% and individual-level genotyping callrate < 95%. Monomorphic SNPs were removed, and there was no filter on HWE or MAF. IMPUTE version 2.1.0 was used to perform imputation for the MESA SHARe Caucasian participants (chromosomes 1-22) using HapMap Phase I and II - CEU as the reference panel (release #24 - NCBI Build 36 (dbSNP b126)). Relationship inference was performed using KING[12] to identify first- and second- degree relatives, and an unrelated set of individuals was identified for genome-wide association analysis.

Fatty acids were obtained for a subset of 2,767 individuals with genotypes available through MESA SHARe, with approximately equal representation from the four ethnic groups (713 Caucasians, 712 Chinese, 645 African Americans, and 697 Hispanics). The fatty acids were measured in EDTA plasma, frozen at –70°C, using methods previously described by Cao et al [4]. Lipids are extracted from the plasma using a chloroform/methanol extraction method and the cholesterol esters, triglyceride, phospholipids and free fatty acids are separated by thin layer chromatography. The fatty acid methyl esters are obtained from the phospholipids and are detected by gas chromatography flame ionization. Individual fatty acids are expressed as a percent of total fatty acids. 28 fatty acids were identified.

**References**

1. (1989) The Atherosclerosis Risk in Communities (ARIC) Study: design and objectives. The ARIC investigators. Am J Epidemiol 129: 687-702.

2. Purcell S, Neale B, Todd-Brown K, Thomas L, Ferreira MA, et al. (2007) PLINK: a tool set for whole-genome association and population-based linkage analyses. Am J Hum Genet 81: 559-75.

3. Price AL, Patterson NJ, Plenge RM, Weinblatt ME, Shadick NA, et al. (2006) Principal components analysis corrects for stratification in genome-wide association studies. Nat Genet 38: 904-9.

4. Cao J, Schwichtenberg KA, Hanson NQ, and Tsai MY (2006) Incorporation and clearance of omega-3 fatty acids in erythrocyte membranes and plasma phospholipids. Clin Chem 52: 2265-72.

5. Fried LP, Borhani NO, Enright P, Furberg CD, Gardin JM, et al. (1991) The Cardiovascular Health Study: design and rationale. Ann Epidemiol 1: 263-76.

6. Friedman GD, Cutter GR, Donahue RP, Hughes GH, Hulley SB, et al. (1988) CARDIA: study design, recruitment, and some characteristics of the examined subjects. J Clin Epidemiol 41: 1105-16.

7. Fornage M and Doris PA (2005) Single-nucleotide polymorphism genotyping for disease association studies. Methods Mol Med 108: 159-72.

8. Ferrucci L, Cherubini A, Bandinelli S, Bartali B, Corsi A, et al. (2006) Relationship of plasma polyunsaturated fatty acids to circulating inflammatory markers. J Clin Endocrinol Metab 91: 439-46.

9. Lepage G and Roy CC (1986) Direct transesterification of all lipids in a one-step reaction. J Lipid Res 27: 114-20.

10. Rodriguez-Palmero M, Lopez-Sabater MC, Castellote-Bargallo AI, De la Torre-Boronat MC, and Rivero-Urgell M (1998) Comparison of two methods for the determination of fatty acid profiles in plasma and erythrocytes. J Chromatogr A 793: 435-40.

11. Bild DE, Bluemke DA, Burke GL, Detrano R, Diez Roux AV, et al. (2002) Multi-ethnic study of atherosclerosis: objectives and design. Am J Epidemiol 156: 871-81.

12. Manichaikul A, Mychaleckyj J, Rich SS, Daly K, Sale M, et al. (2010) Robust relationship inference in genome wide association studies. Bioinformatics In Press.
