# Supplementary material for: Genetic Loci Associated with Plasma Phospholipid n-3 Fatty Acids: A Meta-Analysis of Genome-Wide Association Studies from the CHARGE Consortium
Source: PLoS Genet. 2011 Jul 28;7(7):e1002193. doi: 10.1371/journal.pgen.1002193 (PMC3145614; doi:10.1371/journal.pgen.1002193)
Supplement: Table S1 — Supplementary results from the CHARGE consortium. A. Comprehensive results for ALA with p<5*10−6. B. Comprehensive results for EPA with p<5*10−6. C. Comprehensive results for DPA with p<5*10−6. D. Comprehensive results for DHA with p<5*10−6. E. Results for DPA adjusted for rs2236212 and rs174547 with p<5*10−8. (DOC) [file pgen.1002193.s002.doc]

| **Table S1. Supplementary results from the CHARGE consortium.** | | | | | | | | | | | | | | | | | | | | |  |
| --- | --- | --- | --- | --- | --- | --- | --- | --- | --- | --- | --- | --- | --- | --- | --- | --- | --- | --- | --- | --- | --- |
| **A. Comprehensive results for ALA with p < 5*10^-6^** | | | | | | | | | | | | | | | | | | | | |  |
| MarkerName | Effect allele | | | | | Effect* | | | P.value | | | | Chr | | Position | | Nearest Gene** | | | |  |
| rs174547 | t | | | | | -0.0159 | | | 3.47E-64 | | | | 11 | | 61327359 | | **FADS1** | | | |  |
| rs174550 | t | | | | | -0.0159 | | | 5.61E-64 | | | | 11 | | 61328054 | | **FADS1** | | | |  |
| rs102275 | t | | | | | -0.0158 | | | 7.38E-64 | | | | 11 | | 61314379 | | **C11orf10** | | | |  |
| rs174536 | a | | | | | -0.0159 | | | 1.00E-63 | | | | 11 | | 61308503 | | **C11orf9** | | | |  |
| rs174537 | t | | | | | 0.0159 | | | 1.04E-63 | | | | 11 | | 61309256 | | **C11orf9** | | | |  |
| rs174535 | t | | | | | -0.0159 | | | 1.05E-63 | | | | 11 | | 61307932 | | **C11orf9** | | | |  |
| rs174545 | c | | | | | -0.0158 | | | 1.41E-63 | | | | 11 | | 61325882 | | **FADS1** | | | |  |
| rs174546 | t | | | | | 0.0158 | | | 1.70E-63 | | | | 11 | | 61326406 | | **FADS1** | | | |  |
| rs1535 | a | | | | | -0.0157 | | | 3.00E-63 | | | | 11 | | 61354548 | | **FADS2** | | | |  |
| rs174574 | a | | | | | 0.0156 | | | 2.76E-62 | | | | 11 | | 61356918 | | **FADS2** | | | |  |
| rs174577 | a | | | | | 0.0155 | | | 1.09E-60 | | | | 11 | | 61361390 | | **FADS2** | | | |  |
| rs174576 | a | | | | | 0.0156 | | | 1.17E-60 | | | | 11 | | 61360086 | | **FADS2** | | | |  |
| rs174583 | t | | | | | 0.0155 | | | 2.13E-60 | | | | 11 | | 61366326 | | **FADS2** | | | |  |
| rs174541 | t | | | | | -0.0155 | | | 3.69E-60 | | | | 11 | | 61322484 | | FADS1 | | | |  |
| rs174578 | a | | | | | 0.0155 | | | 4.82E-60 | | | | 11 | | 61362075 | | **FADS2** | | | |  |
| rs4246215 | t | | | | | 0.0154 | | | 8.59E-60 | | | | 11 | | 61320875 | | **FEN1** | | | |  |
| rs174528 | t | | | | | -0.0155 | | | 3.24E-59 | | | | 11 | | 61300075 | | **C11orf9** | | | |  |
| rs174548 | c | | | | | -0.0159 | | | 8.21E-59 | | | | 11 | | 61327924 | | **FADS1** | | | |  |
| rs174549 | a | | | | | 0.0159 | | | 1.88E-58 | | | | 11 | | 61327958 | | **FADS1** | | | |  |
| rs174555 | t | | | | | -0.0158 | | | 4.95E-58 | | | | 11 | | 61336336 | | **FADS1** | | | |  |
| rs174556 | t | | | | | 0.0155 | | | 4.65E-57 | | | | 11 | | 61337211 | | **FADS1** | | | |  |
| rs174601 | t | | | | | 0.0162 | | | 7.15E-57 | | | | 11 | | 61379716 | | **FADS2** | | | |  |
| rs174538 | a | | | | | 0.0154 | | | 1.39E-54 | | | | 11 | | 61316657 | | **C11orf10** | | | |  |
| rs174534 | a | | | | | -0.0147 | | | 2.70E-50 | | | | 11 | | 61306034 | | **C11orf9** | | | |  |
| rs108499 | t | | | | | 0.0148 | | | 4.16E-50 | | | | 11 | | 61303813 | | **C11orf9** | | | |  |
| rs174570 | t | | | | | 0.0157 | | | 9.04E-34 | | | | 11 | | 61353788 | | **FADS2** | | | |  |
| rs174575 | c | | | | | -0.0121 | | | 8.94E-31 | | | | 11 | | 61358579 | | **FADS2** | | | |  |
| rs2727270 | t | | | | | 0.0159 | | | 3.52E-30 | | | | 11 | | 61359813 | | **FADS2** | | | |  |
| rs2727271 | a | | | | | -0.0159 | | | 4.97E-30 | | | | 11 | | 61359934 | | **FADS2** | | | |  |
| rs2524299 | a | | | | | -0.0155 | | | 3.34E-29 | | | | 11 | | 61361358 | | **FADS2** | | | |  |
| rs2072114 | a | | | | | -0.015 | | | 2.41E-28 | | | | 11 | | 61361791 | | **FADS2** | | | |  |
| rs174591 | a | | | | | 0.0116 | | | 1.21E-26 | | | | 11 | | 61374252 | | **FADS2** | | | |  |
| rs174448 | a | | | | | -0.0094 | | | 3.86E-25 | | | | 11 | | 61396149 | | FADS3 | | | |  |
| rs174449 | a | | | | | -0.0094 | | | 7.61E-25 | | | | 11 | | 61396955 | | FADS3 | | | |  |
| rs2845573 | a | | | | | -0.0168 | | | 2.79E-24 | | | | 11 | | 61358484 | | **FADS2** | | | |  |
| rs174455 | a | | | | | -0.0094 | | | 5.90E-24 | | | | 11 | | 61412693 | | **FADS3** | | | |  |
| rs174602 | t | | | | | -0.0158 | | | 1.82E-23 | | | | 11 | | 61380990 | | **FADS2** | | | |  |
| rs509360 | a | | | | | -0.011 | | | 3.22E-23 | | | | 11 | | 61305135 | | **C11orf9** | | | |  |
| rs2851682 | a | | | | | -0.0162 | | | 3.34E-23 | | | | 11 | | 61372588 | | **FADS2** | | | |  |
| rs422249 | t | | | | | 0.0094 | | | 8.27E-23 | | | | 11 | | 61396064 | | FADS3 | | | |  |
| rs2526678 | a | | | | | 0.0172 | | | 1.88E-22 | | | | 11 | | 61380369 | | **FADS2** | | | |  |
| rs174579 | t | | | | | 0.0106 | | | 7.56E-21 | | | | 11 | | 61362189 | | **FADS2** | | | |  |
| rs174532 | a | | | | | -0.0114 | | | 1.31E-20 | | | | 11 | | 61305450 | | **C11orf9** | | | |  |
| rs174593 | t | | | | | -0.0115 | | | 1.37E-19 | | | | 11 | | 61375407 | | **FADS2** | | | |  |
| rs174616 | a | | | | | 0.0079 | | | 1.43E-19 | | | | 11 | | 61385698 | | **FADS2** | | | |  |
| rs174597 | c | | | | | 0.0116 | | | 1.44E-19 | | | | 11 | | 61377616 | | **FADS2** | | | |  |
| rs174585 | a | | | | | 0.0106 | | | 1.46E-19 | | | | 11 | | 61368270 | | **FADS2** | | | |  |
| rs174611 | t | | | | | -0.0088 | | | 2.66E-19 | | | | 11 | | 61384457 | | **FADS2** | | | |  |
| rs149803 | c | | | | | 0.0121 | | | 8.04E-19 | | | | 11 | | 61295596 | | **C11orf9** | | | |  |
| rs174605 | t | | | | | 0.0086 | | | 5.59E-18 | | | | 11 | | 61383497 | | **FADS2** | | | |  |
| rs174450 | t | | | | | -0.0076 | | | 8.27E-18 | | | | 11 | | 61398118 | | **FADS3** | | | |  |
| rs174626 | a | | | | | -0.0074 | | | 5.51E-17 | | | | 11 | | 61393633 | | FADS2 | | | |  |
| rs2269928 | t | | | | | -0.0123 | | | 1.22E-16 | | | | 11 | | 61294105 | | **C11orf9** | | | |  |
| rs174634 | c | | | | | -0.0077 | | | 3.20E-14 | | | | 11 | | 61403963 | | **FADS3** | | | |  |
| rs968567 | t | | | | | 0.0093 | | | 4.43E-14 | | | | 11 | | 61352140 | | FADS2 | | | |  |
| rs174464 | a | | | | | 0.0076 | | | 7.61E-14 | | | | 11 | | 61414502 | | **FADS3** | | | |  |
| rs412334 | t | | | | | -0.0118 | | | 9.72E-14 | | | | 11 | | 61316837 | | **FEN1** | | | |  |
| rs174456 | a | | | | | -0.0076 | | | 1.09E-13 | | | | 11 | | 61412758 | | **FADS3** | | | |  |
| rs1000778 | a | | | | | 0.0074 | | | 1.13E-13 | | | | 11 | | 61411881 | | **FADS3** | | | |  |
| rs174468 | a | | | | | -0.0076 | | | 1.11E-12 | | | | 11 | | 61420267 | | RAB3IL1 | | | |  |
| rs526126 | c | | | | | -0.0105 | | | 1.34E-12 | | | | 11 | | 61381461 | | **FADS2** | | | |  |
| rs174478 | t | | | | | 0.0074 | | | 3.30E-12 | | | | 11 | | 61435152 | | **RAB3IL1** | | | |  |
| rs174476 | t | | | | | -0.0074 | | | 3.48E-12 | | | | 11 | | 61430694 | | **RAB3IL1** | | | |  |
| rs666870 | a | | | | | -0.0074 | | | 3.54E-12 | | | | 11 | | 61434055 | | **RAB3IL1** | | | |  |
| rs174589 | c | | | | | -0.0077 | | | 2.23E-11 | | | | 11 | | 61372379 | | **FADS2** | | | |  |
| rs198464 | a | | | | | -0.0057 | | | 2.48E-11 | | | | 11 | | 61278197 | | C11orf9 | | | |  |
| rs198462 | a | | | | | -0.0057 | | | 3.33E-11 | | | | 11 | | 61280695 | | **C11orf9** | | | |  |
| rs198476 | a | | | | | -0.0058 | | | 3.56E-11 | | | | 11 | | 61282306 | | **C11orf9** | | | |  |
| rs740006 | t | | | | | 0.0163 | | | 1.32E-10 | | | | 11 | | 61314444 | | **C11orf10** | | | |  |
| rs17762402 | a | | | | | -0.0186 | | | 1.56E-10 | | | | 11 | | 61309777 | | **C11orf9** | | | |  |
| rs650436 | t | | | | | -0.0062 | | | 1.87E-10 | | | | 11 | | 61293006 | | **C11orf9** | | | |  |
| rs579383 | a | | | | | 0.0058 | | | 1.02E-09 | | | | 11 | | 61293159 | | **C11orf9** | | | |  |
| rs174627 | a | | | | | 0.0072 | | | 2.62E-09 | | | | 11 | | 61394042 | | FADS2 | | | |  |
| rs174479 | c | | | | | -0.0089 | | | 2.85E-09 | | | | 11 | | 61435330 | | **RAB3IL1** | | | |  |
| rs1692120 | a | | | | | -0.0051 | | | 1.41E-08 | | | | 11 | | 61174048 | | DAGLA | | | |  |
| rs174469 | t | | | | | 0.0114 | | | 7.47E-08 | | | | 11 | | 61424019 | | **RAB3IL1** | | | |  |
| rs2453710 | a | | | | | 0.005 | | | 8.05E-08 | | | | 11 | | 61163118 | | DAGLA | | | |  |
| rs569258 | t | | | | | 0.0049 | | | 8.49E-08 | | | | 11 | | 61277244 | | C11orf9 | | | |  |
| rs17156426 | a | | | | | -0.0121 | | | 1.42E-07 | | | | 11 | | 61365899 | | **FADS2** | | | |  |
| rs17764935 | a | | | | | 0.014 | | | 1.71E-07 | | | | 11 | | 61421333 | | RAB3IL1 | | | |  |
| rs198426 | t | | | | | -0.0047 | | | 2.83E-07 | | | | 11 | | 61247062 | | **DAGLA** | | | |  |
| rs16832011 | a | | | | | 0.0155 | | | 3.15E-07 | | | | 2 | | 136261769 | | LCT | | | |  |
| rs4985167 | t | | | | | 0.0059 | | | 3.16E-07 | | | | 16 | | 14990366 | | **PDXDC1** | | | |  |
| rs17764324 | t | | | | | 0.0076 | | | 6.97E-07 | | | | 11 | | 61391664 | | FADS2 | | | |  |
| rs17831757 | t | | | | | -0.0076 | | | 7.52E-07 | | | | 11 | | 61391776 | | FADS2 | | | |  |
| rs11230815 | c | | | | | -0.0076 | | | 7.94E-07 | | | | 11 | | 61392702 | | FADS2 | | | |  |
| rs7200543 | a | | | | | -0.0048 | | | 8.80E-07 | | | | 16 | | 15037471 | | **PDXDC1** | | | |  |
| rs7104849 | a | | | | | -0.0075 | | | 8.97E-07 | | | | 11 | | 61394620 | | FADS3 | | | |  |
| rs1741 | c | | | | | 0.0048 | | | 1.17E-06 | | | | 16 | | 15037852 | | **PDXDC1** | | | |  |
| rs6498540 | a | | | | | -0.0048 | | | 1.18E-06 | | | | 16 | | 15038095 | | **PDXDC1** | | | |  |
| rs1121 | a | | | | | 0.0048 | | | 1.20E-06 | | | | 16 | | 15038577 | | **PDXDC1** | | | |  |
| rs1135999 | a | | | | | -0.0047 | | | 1.23E-06 | | | | 16 | | 15039463 | | **NTAN1;PDXDC1** | | | |  |
| rs2740 | a | | | | | -0.0047 | | | 1.25E-06 | | | | 16 | | 15039609 | | **NTAN1;PDXDC1** | | | |  |
| rs1136001 | t | | | | | 0.0047 | | | 1.27E-06 | | | | 16 | | 15039475 | | **NTAN1;PDXDC1** | | | |  |
| rs4985124 | t | | | | | -0.0047 | | | 1.29E-06 | | | | 16 | | 15032942 | | **PDXDC1** | | | |  |
| rs16966952 | a | | | | | 0.0047 | | | 1.29E-06 | | | | 16 | | 15043444 | | **NTAN1** | | | |  |
| rs367543 | t | | | | | 0.0052 | | | 1.29E-06 | | | | 8 | | 9071558 | | PPP1R3B | | | |  |
| rs4985148 | a | | | | | -0.0047 | | | 1.46E-06 | | | | 16 | | 15055289 | | **NTAN1** | | | |  |
| rs3803573 | t | | | | | 0.0047 | | | 1.46E-06 | | | | 16 | | 15045914 | | **NTAN1** | | | |  |
| rs4500751 | t | | | | | 0.0047 | | | 1.58E-06 | | | | 16 | | 15047712 | | **NTAN1** | | | |  |
| rs7482316 | a | | | | | -0.0074 | | | 1.59E-06 | | | | 11 | | 61396774 | | FADS3 | | | |  |
| rs1076275 | c | | | | | -0.0047 | | | 1.60E-06 | | | | 8 | | 5461777 | | CSMD1 | | | |  |
| rs16831992 | c | | | | | -0.0133 | | | 1.90E-06 | | | | 2 | | 136225930 | | **UBXD2** | | | |  |
| rs4985155 | a | | | | | -0.0045 | | | 2.05E-06 | | | | 16 | | 15036960 | | **PDXDC1** | | | |  |
| rs6872 | a | | | | | 0.005 | | | 2.98E-06 | | | | 1 | | 26017261 | | **SEPN1** | | | |  |
| rs11230767 | a | | | | | -0.0042 | | | 3.32E-06 | | | | 11 | | 61183508 | | DAGLA | | | |  |
| rs12547955 | t | | | | | 0.0049 | | | 3.57E-06 | | | | 8 | | 9073239 | | PPP1R3B | | | |  |
| rs439989 | c | | | | | -0.005 | | | 3.83E-06 | | | | 8 | | 9071154 | | PPP1R3B | | | |  |
| rs106380 | c | | | | | 0.005 | | | 3.88E-06 | | | | 8 | | 9072488 | | PPP1R3B | | | |  |
| rs7394871 | a | | | | | 0.0129 | | | 4.05E-06 | | | | 11 | | 61409090 | | **FADS3** | | | |  |
| rs4135168 | t | | | | | -0.0048 | | | 4.95E-06 | | | | 9 | | 112056706 | | **TXN** | | | |  |
| **B. Comprehensive results for EPA with p <5*10^-6^** | | | | | | | | | | | | | | | | | | | |  | |
| MarkerName | | Effect allele | | Effect* | | | | P.value | | | Chr | | | | | Position | | Nearest Gene** | |  | |
| rs174538 | | a | | -0.0834 | | | | 5.37E-58 | | | 11 | | | | | 61316657 | | **C11orf10** | |  | |
| rs174535 | | t | | 0.0822 | | | | 6.03E-58 | | | 11 | | | | | 61307932 | | **C11orf9** | |  | |
| rs174536 | | a | | 0.0822 | | | | 6.68E-58 | | | 11 | | | | | 61308503 | | **C11orf9** | |  | |
| rs174537 | | t | | -0.0819 | | | | 1.01E-57 | | | 11 | | | | | 61309256 | | **C11orf9** | |  | |
| rs174550 | | t | | 0.0821 | | | | 1.08E-57 | | | 11 | | | | | 61328054 | | **FADS1** | |  | |
| rs174547 | | t | | 0.082 | | | | 1.83E-57 | | | 11 | | | | | 61327359 | | **FADS1** | |  | |
| rs174546 | | t | | -0.0818 | | | | 2.68E-57 | | | 11 | | | | | 61326406 | | **FADS1** | |  | |
| rs102275 | | t | | 0.0815 | | | | 3.79E-57 | | | 11 | | | | | 61314379 | | **C11orf10** | |  | |
| rs174545 | | c | | 0.0817 | | | | 4.77E-57 | | | 11 | | | | | 61325882 | | **FADS1** | |  | |
| rs174541 | | t | | 0.0812 | | | | 3.11E-55 | | | 11 | | | | | 61322484 | | FADS1 | |  | |
| rs174574 | | a | | -0.0802 | | | | 4.03E-55 | | | 11 | | | | | 61356918 | | **FADS2** | |  | |
| rs4246215 | | t | | -0.0811 | | | | 5.96E-55 | | | 11 | | | | | 61320875 | | **FEN1** | |  | |
| rs1535 | | a | | 0.0798 | | | | 6.46E-55 | | | 11 | | | | | 61354548 | | **FADS2** | |  | |
| rs174556 | | t | | -0.0812 | | | | 6.76E-55 | | | 11 | | | | | 61337211 | | **FADS1** | |  | |
| rs174576 | | a | | -0.0806 | | | | 1.70E-54 | | | 11 | | | | | 61360086 | | **FADS2** | |  | |
| rs174577 | | a | | -0.0804 | | | | 2.44E-54 | | | 11 | | | | | 61361390 | | **FADS2** | |  | |
| rs174578 | | a | | -0.0806 | | | | 2.81E-54 | | | 11 | | | | | 61362075 | | **FADS2** | |  | |
| rs174583 | | t | | -0.0804 | | | | 6.52E-54 | | | 11 | | | | | 61366326 | | **FADS2** | |  | |
| rs174555 | | t | | 0.0812 | | | | 2.15E-53 | | | 11 | | | | | 61336336 | | **FADS1** | |  | |
| rs174601 | | t | | -0.0871 | | | | 4.13E-53 | | | 11 | | | | | 61379716 | | **FADS2** | |  | |
| rs174549 | | a | | -0.0811 | | | | 5.30E-53 | | | 11 | | | | | 61327958 | | **FADS1** | |  | |
| rs174528 | | t | | 0.0787 | | | | 5.50E-53 | | | 11 | | | | | 61300075 | | **C11orf9** | |  | |
| rs174548 | | c | | 0.0809 | | | | 7.85E-53 | | | 11 | | | | | 61327924 | | **FADS1** | |  | |
| rs174534 | | a | | 0.078 | | | | 3.20E-52 | | | 11 | | | | | 61306034 | | **C11orf9** | |  | |
| rs108499 | | t | | -0.0788 | | | | 4.45E-52 | | | 11 | | | | | 61303813 | | **C11orf9** | |  | |
| rs174570 | | t | | -0.0813 | | | | 3.49E-35 | | | 11 | | | | | 61353788 | | **FADS2** | |  | |
| rs2845573 | | a | | 0.093 | | | | 2.19E-32 | | | 11 | | | | | 61358484 | | **FADS2** | |  | |
| rs2851682 | | a | | 0.0906 | | | | 2.83E-32 | | | 11 | | | | | 61372588 | | **FADS2** | |  | |
| rs2526678 | | a | | -0.0988 | | | | 6.84E-32 | | | 11 | | | | | 61380369 | | **FADS2** | |  | |
| rs174575 | | c | | 0.0631 | | | | 4.39E-29 | | | 11 | | | | | 61358579 | | **FADS2** | |  | |
| rs2072114 | | a | | 0.0746 | | | | 1.42E-28 | | | 11 | | | | | 61361791 | | **FADS2** | |  | |
| rs2727270 | | t | | -0.0762 | | | | 5.78E-28 | | | 11 | | | | | 61359813 | | **FADS2** | |  | |
| rs2727271 | | a | | 0.0761 | | | | 6.10E-28 | | | 11 | | | | | 61359934 | | **FADS2** | |  | |
| rs2524299 | | a | | 0.0748 | | | | 6.93E-28 | | | 11 | | | | | 61361358 | | **FADS2** | |  | |
| rs174448 | | a | | 0.0536 | | | | 7.35E-28 | | | 11 | | | | | 61396149 | | FADS3 | |  | |
| rs174449 | | a | | 0.0534 | | | | 1.12E-27 | | | 11 | | | | | 61396955 | | FADS3 | |  | |
| rs174579 | | t | | -0.062 | | | | 2.59E-27 | | | 11 | | | | | 61362189 | | **FADS2** | |  | |
| rs174585 | | a | | -0.0636 | | | | 3.37E-27 | | | 11 | | | | | 61368270 | | **FADS2** | |  | |
| rs422249 | | t | | -0.0547 | | | | 1.61E-26 | | | 11 | | | | | 61396064 | | FADS3 | |  | |
| rs174455 | | a | | 0.0515 | | | | 5.37E-26 | | | 11 | | | | | 61412693 | | **FADS3** | |  | |
| rs174591 | | a | | -0.0626 | | | | 6.18E-26 | | | 11 | | | | | 61374252 | | **FADS2** | |  | |
| rs174605 | | t | | -0.0558 | | | | 8.23E-25 | | | 11 | | | | | 61383497 | | **FADS2** | |  | |
| rs174611 | | t | | 0.055 | | | | 1.03E-24 | | | 11 | | | | | 61384457 | | **FADS2** | |  | |
| rs174593 | | t | | 0.0665 | | | | 8.36E-24 | | | 11 | | | | | 61375407 | | **FADS2** | |  | |
| rs174597 | | c | | -0.0666 | | | | 8.72E-24 | | | 11 | | | | | 61377616 | | **FADS2** | |  | |
| rs174602 | | t | | 0.0824 | | | | 5.27E-23 | | | 11 | | | | | 61380990 | | **FADS2** | |  | |
| rs174589 | | c | | 0.0585 | | | | 5.88E-23 | | | 11 | | | | | 61372379 | | **FADS2** | |  | |
| rs174616 | | a | | -0.0479 | | | | 4.99E-22 | | | 11 | | | | | 61385698 | | **FADS2** | |  | |
| rs968567 | | t | | -0.0588 | | | | 3.79E-21 | | | 11 | | | | | 61352140 | | FADS2 | |  | |
| rs174626 | | a | | 0.0457 | | | | 6.06E-20 | | | 11 | | | | | 61393633 | | FADS2 | |  | |
| rs174450 | | t | | 0.0457 | | | | 4.97E-19 | | | 11 | | | | | 61398118 | | **FADS3** | |  | |
| rs174532 | | a | | 0.0574 | | | | 4.72E-18 | | | 11 | | | | | 61305450 | | **C11orf9** | |  | |
| rs526126 | | c | | 0.0674 | | | | 9.62E-18 | | | 11 | | | | | 61381461 | | **FADS2** | |  | |
| rs174468 | | a | | 0.05 | | | | 2.14E-17 | | | 11 | | | | | 61420267 | | RAB3IL1 | |  | |
| rs666870 | | a | | 0.0491 | | | | 1.17E-16 | | | 11 | | | | | 61434055 | | **RAB3IL1** | |  | |
| rs174476 | | t | | 0.0491 | | | | 1.21E-16 | | | 11 | | | | | 61430694 | | **RAB3IL1** | |  | |
| rs174478 | | t | | -0.049 | | | | 1.23E-16 | | | 11 | | | | | 61435152 | | **RAB3IL1** | |  | |
| rs174464 | | a | | -0.043 | | | | 5.23E-15 | | | 11 | | | | | 61414502 | | **FADS3** | |  | |
| rs174456 | | a | | 0.0428 | | | | 5.77E-15 | | | 11 | | | | | 61412758 | | **FADS3** | |  | |
| rs174634 | | c | | 0.0421 | | | | 5.80E-15 | | | 11 | | | | | 61403963 | | **FADS3** | |  | |
| rs2269928 | | t | | 0.0639 | | | | 7.13E-15 | | | 11 | | | | | 61294105 | | **C11orf9** | |  | |
| rs149803 | | c | | -0.0626 | | | | 7.24E-15 | | | 11 | | | | | 61295596 | | **C11orf9** | |  | |
| rs1000778 | | a | | -0.0409 | | | | 6.19E-14 | | | 11 | | | | | 61411881 | | **FADS3** | |  | |
| rs509360 | | a | | 0.0448 | | | | 1.16E-13 | | | 11 | | | | | 61305135 | | **C11orf9** | |  | |
| rs174479 | | c | | 0.0548 | | | | 1.27E-13 | | | 11 | | | | | 61435330 | | **RAB3IL1** | |  | |
| rs174627 | | a | | -0.0475 | | | | 9.53E-13 | | | 11 | | | | | 61394042 | | FADS2 | |  | |
| rs7394871 | | a | | -0.0912 | | | | 1.13E-12 | | | 11 | | | | | 61409090 | | **FADS3** | |  | |
| rs3798713 | | c | | 0.035 | | | | 1.93E-12 | | | 6 | | | | | 11116608 | | **ELOVL2** | |  | |
| rs2236212 | | c | | 0.0351 | | | | 1.97E-12 | | | 6 | | | | | 11103001 | | **ELOVL2** | |  | |
| rs3734398 | | t | | -0.0352 | | | | 3.99E-12 | | | 6 | | | | | 11090959 | | **ELOVL2** | |  | |
| rs1321536 | | t | | -0.0339 | | | | 1.07E-11 | | | 6 | | | | | 11126798 | | **ELOVL2** | |  | |
| rs1323739 | | c | | -0.0337 | | | | 1.25E-11 | | | 6 | | | | | 11112547 | | **ELOVL2** | |  | |
| rs1225737 | | t | | 0.034 | | | | 1.52E-11 | | | 6 | | | | | 11090638 | | **ELOVL2** | |  | |
| rs1570069 | | a | | -0.0333 | | | | 1.68E-11 | | | 6 | | | | | 11125811 | | **ELOVL2** | |  | |
| rs7743830 | | a | | -0.0333 | | | | 1.72E-11 | | | 6 | | | | | 11122206 | | **ELOVL2** | |  | |
| rs3798711 | | t | | -0.0333 | | | | 1.81E-11 | | | 6 | | | | | 11110796 | | **ELOVL2** | |  | |
| rs953413 | | a | | 0.0332 | | | | 1.81E-11 | | | 6 | | | | | 11120845 | | **ELOVL2** | |  | |
| rs2295602 | | t | | -0.0332 | | | | 1.83E-11 | | | 6 | | | | | 11113828 | | **ELOVL2** | |  | |
| rs3798707 | | t | | 0.0334 | | | | 1.87E-11 | | | 6 | | | | | 11099921 | | **ELOVL2** | |  | |
| rs17675322 | | a | | 0.0363 | | | | 1.87E-11 | | | 6 | | | | | 11167171 | | ELOVL2 | |  | |
| rs4532436 | | c | | -0.0338 | | | | 2.06E-11 | | | 6 | | | | | 11091957 | | **ELOVL2** | |  | |
| rs6900220 | | t | | -0.0364 | | | | 2.79E-11 | | | 6 | | | | | 11173124 | | ELOVL2 | |  | |
| rs9295764 | | a | | -0.0352 | | | | 3.31E-11 | | | 6 | | | | | 11153182 | | ELOVL2 | |  | |
| rs9295763 | | c | | 0.0351 | | | | 3.31E-11 | | | 6 | | | | | 11153178 | | ELOVL2 | |  | |
| rs1321535 | | t | | -0.0371 | | | | 4.51E-11 | | | 6 | | | | | 11184012 | | HERV-FRD | |  | |
| rs4713165 | | t | | -0.037 | | | | 4.84E-11 | | | 6 | | | | | 11182288 | | HERV-FRD | |  | |
| rs12665478 | | a | | 0.037 | | | | 5.34E-11 | | | 6 | | | | | 11188811 | | HERV-FRD | |  | |
| rs12526913 | | a | | -0.0372 | | | | 5.44E-11 | | | 6 | | | | | 11190910 | | HERV-FRD | |  | |
| rs4713169 | | c | | 0.0372 | | | | 5.59E-11 | | | 6 | | | | | 11192540 | | HERV-FRD | |  | |
| rs2147041 | | a | | -0.035 | | | | 1.45E-10 | | | 6 | | | | | 11158509 | | ELOVL2 | |  | |
| rs17764935 | | a | | -0.081 | | | | 1.48E-10 | | | 11 | | | | | 61421333 | | RAB3IL1 | |  | |
| rs4711171 | | t | | 0.036 | | | | 1.56E-10 | | | 6 | | | | | 11182333 | | HERV-FRD | |  | |
| rs17764324 | | t | | -0.0483 | | | | 3.73E-10 | | | 11 | | | | | 61391664 | | FADS2 | |  | |
| rs17831757 | | t | | 0.0482 | | | | 3.92E-10 | | | 11 | | | | | 61391776 | | FADS2 | |  | |
| rs11230815 | | c | | 0.0481 | | | | 4.04E-10 | | | 11 | | | | | 61392702 | | FADS2 | |  | |
| rs8523 | | a | | 0.0334 | | | | 4.06E-10 | | | 6 | | | | | 11089039 | | **ELOVL2** | |  | |
| rs7104849 | | a | | 0.0479 | | | | 4.59E-10 | | | 11 | | | | | 61394620 | | FADS3 | |  | |
| rs9393915 | | t | | 0.0389 | | | | 6.55E-10 | | | 6 | | | | | 11180308 | | ELOVL2 | |  | |
| rs3756963 | | t | | -0.0364 | | | | 8.33E-10 | | | 6 | | | | | 11130140 | | **ELOVL2** | |  | |
| rs2180725 | | t | | -0.0361 | | | | 1.08E-09 | | | 6 | | | | | 11133406 | | **ELOVL2** | |  | |
| rs9295757 | | t | | 0.0361 | | | | 1.14E-09 | | | 6 | | | | | 11141611 | | **ELOVL2** | |  | |
| rs2295601 | | a | | 0.0359 | | | | 1.18E-09 | | | 6 | | | | | 11113672 | | **ELOVL2** | |  | |
| rs9368564 | | a | | -0.0375 | | | | 1.28E-09 | | | 6 | | | | | 11168269 | | ELOVL2 | |  | |
| rs17606561 | | a | | 0.0365 | | | | 1.35E-09 | | | 6 | | | | | 11090345 | | **ELOVL2** | |  | |
| rs2281591 | | a | | -0.0359 | | | | 1.41E-09 | | | 6 | | | | | 11098479 | | **ELOVL2** | |  | |
| rs3798719 | | t | | 0.0352 | | | | 1.49E-09 | | | 6 | | | | | 11144811 | | **ELOVL2** | |  | |
| rs174469 | | t | | -0.0573 | | | | 1.63E-09 | | | 11 | | | | | 61424019 | | **RAB3IL1** | |  | |
| rs7482316 | | a | | 0.0452 | | | | 1.87E-09 | | | 11 | | | | | 61396774 | | FADS3 | |  | |
| rs9393903 | | a | | 0.0353 | | | | 2.25E-09 | | | 6 | | | | | 11150895 | | **ELOVL2** | |  | |
| rs3798723 | | a | | 0.0348 | | | | 2.74E-09 | | | 6 | | | | | 11149706 | | **ELOVL2** | |  | |
| rs1109748 | | a | | -0.0535 | | | | 5.46E-09 | | | 11 | | | | | 61479221 | | **BEST1** | |  | |
| rs7744440 | | t | | -0.0334 | | | | 9.62E-09 | | | 6 | | | | | 11146497 | | **ELOVL2** | |  | |
| rs2521572 | | t | | -0.0614 | | | | 1.20E-08 | | | 11 | | | | | 61468051 | | BEST1 | |  | |
| rs3798721 | | a | | -0.0328 | | | | 1.56E-08 | | | 6 | | | | | 11148169 | | **ELOVL2** | |  | |
| rs11230874 | | t | | 0.067 | | | | 1.72E-08 | | | 11 | | | | | 61552162 | | FTH1 | |  | |
| rs3798722 | | a | | -0.0327 | | | | 2.52E-08 | | | 6 | | | | | 11148409 | | **ELOVL2** | |  | |
| rs6918936 | | c | | 0.0296 | | | | 3.34E-08 | | | 6 | | | | | 11068144 | | **SYCP2L** | |  | |
| rs6920247 | | c | | -0.0296 | | | | 3.35E-08 | | | 6 | | | | | 11068159 | | **SYCP2L** | |  | |
| rs4711146 | | t | | -0.0296 | | | | 3.37E-08 | | | 6 | | | | | 11069391 | | **SYCP2L** | |  | |
| rs2295600 | | a | | -0.0295 | | | | 3.42E-08 | | | 6 | | | | | 11070236 | | **SYCP2L** | |  | |
| rs4713103 | | t | | 0.0293 | | | | 3.76E-08 | | | 6 | | | | | 11077127 | | **SYCP2L** | |  | |
| rs9295733 | | a | | 0.0293 | | | | 4.00E-08 | | | 6 | | | | | 11073433 | | **SYCP2L** | |  | |
| rs1225717 | | a | | -0.0292 | | | | 4.21E-08 | | | 6 | | | | | 11086226 | | ELOVL2 | |  | |
| rs412334 | | t | | 0.044 | | | | 4.59E-08 | | | 11 | | | | | 61316837 | | **FEN1** | |  | |
| rs7774711 | | a | | -0.0291 | | | | 4.61E-08 | | | 6 | | | | | 11073007 | | **SYCP2L** | |  | |
| rs498793 | | t | | 0.0351 | | | | 5.11E-08 | | | 11 | | | | | 61381281 | | **FADS2** | |  | |
| rs1145652 | | a | | 0.0356 | | | | 8.39E-08 | | | 5 | | | | | 164696665 | | MAT2B | |  | |
| rs2327325 | | a | | 0.0325 | | | | 1.38E-07 | | | 6 | | | | | 11073640 | | **SYCP2L** | |  | |
| rs740006 | | t | | -0.0655 | | | | 1.82E-07 | | | 11 | | | | | 61314444 | | **C11orf10** | |  | |
| rs174472 | | a | | 0.0328 | | | | 1.98E-07 | | | 11 | | | | | 61428532 | | **RAB3IL1** | |  | |
| rs9983044 | | c | | 0.0728 | | | | 2.27E-07 | | | 21 | | | | | 35338202 | | **RUNX1** | |  | |
| rs1514178 | | t | | 0.0678 | | | | 2.29E-07 | | | 1 | | | | | 60978057 | | NFIA | |  | |
| rs9791208 | | t | | -0.0316 | | | | 2.33E-07 | | | 6 | | | | | 11069635 | | **SYCP2L** | |  | |
| rs10897208 | | a | | 0.0599 | | | | 2.40E-07 | | | 11 | | | | | 61548463 | | FTH1 | |  | |
| rs17674802 | | a | | 0.0314 | | | | 2.43E-07 | | | 6 | | | | | 11077505 | | **SYCP2L** | |  | |
| rs9348766 | | t | | 0.0314 | | | | 2.58E-07 | | | 6 | | | | | 11076568 | | **SYCP2L** | |  | |
| rs4963444 | | a | | -0.059 | | | | 2.76E-07 | | | 11 | | | | | 61529918 | | FTH1 | |  | |
| rs9368506 | | t | | 0.0315 | | | | 2.87E-07 | | | 6 | | | | | 11066458 | | **SYCP2L** | |  | |
| rs7102974 | | t | | -0.1382 | | | | 3.03E-07 | | | 11 | | | | | 61316611 | | **C11orf10** | |  | |
| rs17762402 | | a | | 0.0869 | | | | 5.41E-07 | | | 11 | | | | | 61309777 | | **C11orf9** | |  | |
| rs2585617 | | a | | -0.0312 | | | | 5.95E-07 | | | 6 | | | | | 69394508 | | BAI3 | |  | |
| rs12459897 | | t | | -0.094 | | | | 8.16E-07 | | | 19 | | | | | 36288618 | | TSHZ3 | |  | |
| rs10847425 | | a | | 0.0296 | | | | 1.01E-06 | | | 12 | | | | | 126700427 | | SLC15A4 | |  | |
| rs7264749 | | t | | -0.0711 | | | | 1.02E-06 | | | 20 | | | | | 60821448 | | **NTSR1** | |  | |
| rs7773173 | | c | | 0.0245 | | | | 1.10E-06 | | | 6 | | | | | 11064689 | | **SYCP2L** | |  | |
| rs1960136 | | t | | -0.0291 | | | | 1.26E-06 | | | 12 | | | | | 126645373 | | SLC15A4 | |  | |
| rs1225753 | | a | | -0.0243 | | | | 1.27E-06 | | | 6 | | | | | 11064675 | | **SYCP2L** | |  | |
| rs7626880 | | a | | 0.0818 | | | | 1.36E-06 | | | 3 | | | | | 165896424 | | SI | |  | |
| rs5749970 | | t | | -0.0632 | | | | 1.52E-06 | | | 22 | | | | | 33560810 | | ISX | |  | |
| rs6870738 | | a | | -0.1088 | | | | 1.57E-06 | | | 5 | | | | | 7858774 | | **ADCY2** | |  | |
| rs1225744 | | t | | -0.024 | | | | 1.57E-06 | | | 6 | | | | | 11062526 | | **SYCP2L** | |  | |
| rs3007728 | | t | | -0.0629 | | | | 1.67E-06 | | | 1 | | | | | 18666828 | | KLHDC7A | |  | |
| rs11033114 | | a | | -0.0947 | | | | 1.69E-06 | | | 11 | | | | | 35390200 | | **SLC1A2** | |  | |
| rs11033115 | | a | | 0.0969 | | | | 1.71E-06 | | | 11 | | | | | 35392009 | | **SLC1A2** | |  | |
| rs4499314 | | t | | -0.0761 | | | | 1.88E-06 | | | 18 | | | | | 26165113 | | DSC3 | |  | |
| rs7480288 | | t | | 0.0411 | | | | 1.93E-06 | | | 11 | | | | | 121230316 | | SORL1 | |  | |
| rs941435 | | t | | 0.0593 | | | | 1.99E-06 | | | 22 | | | | | 33560303 | | ISX | |  | |
| rs10484792 | | c | | -0.0305 | | | | 2.03E-06 | | | 6 | | | | | 69523964 | | **BAI3** | |  | |
| rs1403920 | | a | | 0.0308 | | | | 2.06E-06 | | | 6 | | | | | 69531393 | | **BAI3** | |  | |
| rs13220538 | | a | | 0.0308 | | | | 2.08E-06 | | | 6 | | | | | 69531860 | | **BAI3** | |  | |
| rs10512289 | | t | | 0.076 | | | | 2.27E-06 | | | 9 | | | | | 103550279 | | GRIN3A | |  | |
| rs7955165 | | t | | 0.0252 | | | | 2.28E-06 | | | 12 | | | | | 126656880 | | SLC15A4 | |  | |
| rs2421897 | | c | | -0.0959 | | | | 2.28E-06 | | | 11 | | | | | 35394439 | | **SLC1A2** | |  | |
| rs10847417 | | t | | 0.0251 | | | | 2.38E-06 | | | 12 | | | | | 126658210 | | SLC15A4 | |  | |
| rs7076844 | | a | | -0.0261 | | | | 2.40E-06 | | | 10 | | | | | 19866244 | | PLXDC2 | |  | |
| rs10847423 | | t | | -0.0293 | | | | 2.51E-06 | | | 12 | | | | | 126695202 | | SLC15A4 | |  | |
| rs12792401 | | t | | -0.0412 | | | | 2.57E-06 | | | 11 | | | | | 121243872 | | SORL1 | |  | |
| rs4406722 | | c | | -0.0291 | | | | 2.57E-06 | | | 10 | | | | | 19941019 | | PLXDC2 | |  | |
| rs4691309 | | a | | -0.048 | | | | 2.57E-06 | | | 4 | | | | | 157340664 | | CTSO | |  | |
| rs17125867 | | t | | -0.0402 | | | | 2.58E-06 | | | 11 | | | | | 121217313 | | SORL1 | |  | |
| rs7207094 | | a | | -0.0325 | | | | 2.72E-06 | | | 17 | | | | | 29749782 | | CCL1 | |  | |
| rs1225741 | | a | | -0.0239 | | | | 2.76E-06 | | | 6 | | | | | 11060229 | | **SYCP2L** | |  | |
| rs4760993 | | a | | 0.0251 | | | | 2.87E-06 | | | 12 | | | | | 126678030 | | SLC15A4 | |  | |
| rs1414642 | | t | | -0.0619 | | | | 2.88E-06 | | | 1 | | | | | 18660033 | | KLHDC7A | |  | |
| rs3852471 | | a | | 0.0266 | | | | 2.88E-06 | | | 10 | | | | | 19862319 | | PLXDC2 | |  | |
| rs1007323 | | t | | 0.0239 | | | | 2.94E-06 | | | 6 | | | | | 11061230 | | **SYCP2L** | |  | |
| rs17125870 | | t | | 0.0411 | | | | 3.03E-06 | | | 11 | | | | | 121246154 | | SORL1 | |  | |
| rs4760949 | | a | | -0.025 | | | | 3.13E-06 | | | 12 | | | | | 126677892 | | SLC15A4 | |  | |
| rs7946844 | | a | | 0.0413 | | | | 3.15E-06 | | | 11 | | | | | 121247877 | | SORL1 | |  | |
| rs2717298 | | c | | 0.0898 | | | | 3.15E-06 | | | 3 | | | | | 183880984 | | ATP11B | |  | |
| rs16862008 | | a | | -0.0612 | | | | 3.33E-06 | | | 1 | | | | | 18674073 | | KLHDC7A | |  | |
| rs7947574 | | t | | -0.0414 | | | | 3.42E-06 | | | 11 | | | | | 121248430 | | SORL1 | |  | |
| rs11888148 | | a | | 0.0882 | | | | 3.56E-06 | | | 2 | | | | | 196955819 | | **HECW2** | |  | |
| rs2134485 | | a | | -0.0248 | | | | 3.56E-06 | | | 12 | | | | | 126661423 | | SLC15A4 | |  | |
| rs1033521 | | t | | 0.0305 | | | | 4.05E-06 | | | 6 | | | | | 69561582 | | **BAI3** | |  | |
| rs2242455 | | t | | -0.0269 | | | | 4.10E-06 | | | 12 | | | | | 126699237 | | SLC15A4 | |  | |
| rs7666177 | | c | | 0.0851 | | | | 4.18E-06 | | | 4 | | | | | 2264431 | | **ZFYVE28** | |  | |
| rs17276460 | | t | | -0.1009 | | | | 4.23E-06 | | | 19 | | | | | 14806948 | | OR7A10 | |  | |
| rs17047227 | | t | | -0.0719 | | | | 4.40E-06 | | | 1 | | | | | 216265018 | | SPATA17 | |  | |
| rs650436 | | t | | 0.029 | | | | 4.42E-06 | | | 11 | | | | | 61293006 | | **C11orf9** | |  | |
| rs17818992 | | a | | 0.0806 | | | | 4.43E-06 | | | 4 | | | | | 2253421 | | **ZFYVE28** | |  | |
| rs2542749 | | a | | 0.0413 | | | | 4.45E-06 | | | 18 | | | | | 26385123 | | DSC3 | |  | |
| rs2727266 | | a | | 0.04 | | | | 4.48E-06 | | | 11 | | | | | 61460910 | | BEST1 | |  | |
| rs2521568 | | c | | -0.04 | | | | 4.50E-06 | | | 11 | | | | | 61457509 | | RAB3IL1 | |  | |
| rs6450352 | | a | | 0.0436 | | | | 4.77E-06 | | | 5 | | | | | 55144489 | | **DDX4** | |  | |
| rs11059295 | | a | | 0.0269 | | | | 4.77E-06 | | | 12 | | | | | 126700122 | | SLC15A4 | |  | |
| rs3852482 | | t | | -0.0284 | | | | 4.82E-06 | | | 10 | | | | | 19937200 | | PLXDC2 | |  | |
| rs276197 | | a | | -0.0761 | | | | 4.90E-06 | | | 19 | | | | | 36321625 | | TSHZ3 | |  | |
| rs10764059 | | t | | 0.0257 | | | | 4.95E-06 | | | 10 | | | | | 19871964 | | PLXDC2 | |  | |
| rs7660280 | | c | | -0.0846 | | | | 5.00E-06 | | | 4 | | | | | 2266170 | | **ZFYVE28** | |  | |
| **C. Comprehensive results for DPA with p <5*10^-6^** | | | | | | | | | | | | | | | | | | | | |  |
| MarkerName | | | Effect allele | | Effect* | | | P.value | | | | Chr | | | | Position | | | Nearest Gene ** | |  |
| rs174547 | | | t | | 0.0746 | | | 3.79E-154 | | | | 11 | | | | 61327359 | | | **FADS1** | |  |
| rs174550 | | | t | | 0.0746 | | | 1.69E-153 | | | | 11 | | | | 61328054 | | | **FADS1** | |  |
| rs174546 | | | t | | -0.0744 | | | 2.51E-153 | | | | 11 | | | | 61326406 | | | **FADS1** | |  |
| rs102275 | | | t | | 0.0744 | | | 8.39E-153 | | | | 11 | | | | 61314379 | | | **C11orf10** | |  |
| rs174545 | | | c | | 0.0743 | | | 2.42E-152 | | | | 11 | | | | 61325882 | | | **FADS1** | |  |
| rs1535 | | | a | | 0.0744 | | | 2.55E-152 | | | | 11 | | | | 61354548 | | | **FADS2** | |  |
| rs174535 | | | t | | 0.0746 | | | 1.46E-151 | | | | 11 | | | | 61307932 | | | **C11orf9** | |  |
| rs174536 | | | a | | 0.0745 | | | 1.57E-151 | | | | 11 | | | | 61308503 | | | **C11orf9** | |  |
| rs174574 | | | a | | -0.0745 | | | 1.93E-151 | | | | 11 | | | | 61356918 | | | **FADS2** | |  |
| rs174576 | | | a | | -0.0752 | | | 9.13E-151 | | | | 11 | | | | 61360086 | | | **FADS2** | |  |
| rs174537 | | | t | | -0.0742 | | | 2.11E-150 | | | | 11 | | | | 61309256 | | | **C11orf9** | |  |
| rs174578 | | | a | | -0.0751 | | | 1.94E-149 | | | | 11 | | | | 61362075 | | | **FADS2** | |  |
| rs174577 | | | a | | -0.0747 | | | 2.67E-149 | | | | 11 | | | | 61361390 | | | **FADS2** | |  |
| rs174583 | | | t | | -0.0743 | | | 4.17E-147 | | | | 11 | | | | 61366326 | | | **FADS2** | |  |
| rs174541 | | | t | | 0.0724 | | | 2.30E-140 | | | | 11 | | | | 61322484 | | | FADS1 | |  |
| rs174549 | | | a | | -0.0744 | | | 1.30E-139 | | | | 11 | | | | 61327958 | | | **FADS1** | |  |
| rs4246215 | | | t | | -0.0721 | | | 1.38E-139 | | | | 11 | | | | 61320875 | | | **FEN1** | |  |
| rs174555 | | | t | | 0.0739 | | | 8.76E-139 | | | | 11 | | | | 61336336 | | | **FADS1** | |  |
| rs174556 | | | t | | -0.0729 | | | 1.04E-138 | | | | 11 | | | | 61337211 | | | **FADS1** | |  |
| rs174548 | | | c | | 0.0737 | | | 3.47E-138 | | | | 11 | | | | 61327924 | | | **FADS1** | |  |
| rs174601 | | | t | | -0.078 | | | 2.39E-136 | | | | 11 | | | | 61379716 | | | **FADS2** | |  |
| rs174538 | | | a | | -0.0738 | | | 4.43E-136 | | | | 11 | | | | 61316657 | | | **C11orf10** | |  |
| rs174528 | | | t | | 0.0718 | | | 1.13E-135 | | | | 11 | | | | 61300075 | | | **C11orf9** | |  |
| rs174534 | | | a | | 0.0702 | | | 8.26E-122 | | | | 11 | | | | 61306034 | | | **C11orf9** | |  |
| rs108499 | | | t | | -0.0706 | | | 2.52E-121 | | | | 11 | | | | 61303813 | | | **C11orf9** | |  |
| rs174575 | | | c | | 0.0617 | | | 2.17E-84 | | | | 11 | | | | 61358579 | | | **FADS2** | |  |
| rs174591 | | | a | | -0.0605 | | | 1.18E-73 | | | | 11 | | | | 61374252 | | | **FADS2** | |  |
| rs2072114 | | | a | | 0.0655 | | | 4.45E-64 | | | | 11 | | | | 61361791 | | | **FADS2** | |  |
| rs174579 | | | t | | -0.0573 | | | 4.08E-63 | | | | 11 | | | | 61362189 | | | **FADS2** | |  |
| rs2727270 | | | t | | -0.0658 | | | 1.66E-61 | | | | 11 | | | | 61359813 | | | **FADS2** | |  |
| rs2727271 | | | a | | 0.0657 | | | 2.29E-61 | | | | 11 | | | | 61359934 | | | **FADS2** | |  |
| rs174448 | | | a | | 0.0474 | | | 2.89E-60 | | | | 11 | | | | 61396149 | | | FADS3 | |  |
| rs174449 | | | a | | 0.0472 | | | 7.58E-60 | | | | 11 | | | | 61396955 | | | FADS3 | |  |
| rs174570 | | | t | | -0.0616 | | | 8.12E-60 | | | | 11 | | | | 61353788 | | | **FADS2** | |  |
| rs2524299 | | | a | | 0.0643 | | | 9.19E-60 | | | | 11 | | | | 61361358 | | | **FADS2** | |  |
| rs174585 | | | a | | -0.0579 | | | 3.93E-59 | | | | 11 | | | | 61368270 | | | **FADS2** | |  |
| rs174593 | | | t | | 0.0616 | | | 9.40E-57 | | | | 11 | | | | 61375407 | | | **FADS2** | |  |
| rs174597 | | | c | | -0.0618 | | | 1.12E-56 | | | | 11 | | | | 61377616 | | | **FADS2** | |  |
| rs174602 | | | t | | 0.0784 | | | 1.46E-56 | | | | 11 | | | | 61380990 | | | **FADS2** | |  |
| rs422249 | | | t | | -0.0482 | | | 1.90E-56 | | | | 11 | | | | 61396064 | | | FADS3 | |  |
| rs174455 | | | a | | 0.0462 | | | 9.33E-56 | | | | 11 | | | | 61412693 | | | **FADS3** | |  |
| rs2845573 | | | a | | 0.0697 | | | 4.45E-51 | | | | 11 | | | | 61358484 | | | **FADS2** | |  |
| rs174532 | | | a | | 0.0594 | | | 5.19E-51 | | | | 11 | | | | 61305450 | | | **C11orf9** | |  |
| rs968567 | | | t | | -0.0558 | | | 6.15E-51 | | | | 11 | | | | 61352140 | | | FADS2 | |  |
| rs174616 | | | a | | -0.0414 | | | 6.34E-50 | | | | 11 | | | | 61385698 | | | **FADS2** | |  |
| rs174611 | | | t | | 0.0455 | | | 2.91E-48 | | | | 11 | | | | 61384457 | | | **FADS2** | |  |
| rs174589 | | | c | | 0.0508 | | | 6.47E-48 | | | | 11 | | | | 61372379 | | | **FADS2** | |  |
| rs174450 | | | t | | 0.0405 | | | 6.44E-47 | | | | 11 | | | | 61398118 | | | **FADS3** | |  |
| rs174605 | | | t | | -0.0452 | | | 7.48E-47 | | | | 11 | | | | 61383497 | | | **FADS2** | |  |
| rs2526678 | | | a | | -0.0703 | | | 1.16E-45 | | | | 11 | | | | 61380369 | | | **FADS2** | |  |
| rs174626 | | | a | | 0.0391 | | | 5.63E-44 | | | | 11 | | | | 61393633 | | | FADS2 | |  |
| rs3734398 | | | t | | -0.0404 | | | 9.61E-44 | | | | 6 | | | | 11090959 | | | **ELOVL2** | |  |
| rs2851682 | | | a | | 0.064 | | | 1.83E-43 | | | | 11 | | | | 61372588 | | | **FADS2** | |  |
| rs2236212 | | | c | | 0.0395 | | | 2.87E-43 | | | | 6 | | | | 11103001 | | | **ELOVL2** | |  |
| rs1225737 | | | t | | 0.0396 | | | 7.06E-43 | | | | 6 | | | | 11090638 | | | **ELOVL2** | |  |
| rs4532436 | | | c | | -0.0396 | | | 7.78E-43 | | | | 6 | | | | 11091957 | | | **ELOVL2** | |  |
| rs3798713 | | | c | | 0.0392 | | | 9.71E-43 | | | | 6 | | | | 11116608 | | | **ELOVL2** | |  |
| rs3798707 | | | t | | 0.039 | | | 1.19E-42 | | | | 6 | | | | 11099921 | | | **ELOVL2** | |  |
| rs1321536 | | | t | | -0.0392 | | | 1.25E-42 | | | | 6 | | | | 11126798 | | | **ELOVL2** | |  |
| rs3798711 | | | t | | -0.0387 | | | 2.47E-42 | | | | 6 | | | | 11110796 | | | **ELOVL2** | |  |
| rs1570069 | | | a | | -0.0386 | | | 3.48E-42 | | | | 6 | | | | 11125811 | | | **ELOVL2** | |  |
| rs2295602 | | | t | | -0.0386 | | | 3.56E-42 | | | | 6 | | | | 11113828 | | | **ELOVL2** | |  |
| rs7743830 | | | a | | -0.0386 | | | 4.07E-42 | | | | 6 | | | | 11122206 | | | **ELOVL2** | |  |
| rs953413 | | | a | | 0.0383 | | | 9.07E-42 | | | | 6 | | | | 11120845 | | | **ELOVL2** | |  |
| rs17675322 | | | a | | 0.0422 | | | 3.30E-41 | | | | 6 | | | | 11167171 | | | ELOVL2 | |  |
| rs2269928 | | | t | | 0.0605 | | | 6.19E-41 | | | | 11 | | | | 61294105 | | | **C11orf9** | |  |
| rs9295763 | | | c | | 0.041 | | | 6.64E-41 | | | | 6 | | | | 11153178 | | | ELOVL2 | |  |
| rs9295764 | | | a | | -0.0411 | | | 6.88E-41 | | | | 6 | | | | 11153182 | | | ELOVL2 | |  |
| rs9368564 | | | a | | -0.0481 | | | 1.00E-40 | | | | 6 | | | | 11168269 | | | ELOVL2 | |  |
| rs6900220 | | | t | | -0.0422 | | | 1.21E-40 | | | | 6 | | | | 11173124 | | | ELOVL2 | |  |
| rs526126 | | | c | | 0.062 | | | 3.42E-40 | | | | 11 | | | | 61381461 | | | **FADS2** | |  |
| rs9393915 | | | t | | 0.0482 | | | 6.09E-40 | | | | 6 | | | | 11180308 | | | ELOVL2 | |  |
| rs174634 | | | c | | 0.0422 | | | 6.13E-40 | | | | 11 | | | | 61403963 | | | **FADS3** | |  |
| rs149803 | | | c | | -0.0592 | | | 7.47E-40 | | | | 11 | | | | 61295596 | | | **C11orf9** | |  |
| rs174464 | | | a | | -0.0422 | | | 1.65E-39 | | | | 11 | | | | 61414502 | | | **FADS3** | |  |
| rs2147041 | | | a | | -0.0415 | | | 2.01E-39 | | | | 6 | | | | 11158509 | | | ELOVL2 | |  |
| rs174456 | | | a | | 0.0419 | | | 2.72E-39 | | | | 11 | | | | 61412758 | | | **FADS3** | |  |
| rs17606561 | | | a | | 0.0451 | | | 2.84E-39 | | | | 6 | | | | 11090345 | | | **ELOVL2** | |  |
| rs9393903 | | | a | | 0.0442 | | | 3.19E-39 | | | | 6 | | | | 11150895 | | | **ELOVL2** | |  |
| rs1000778 | | | a | | -0.0415 | | | 3.49E-39 | | | | 11 | | | | 61411881 | | | **FADS3** | |  |
| rs8523 | | | a | | 0.0397 | | | 8.17E-39 | | | | 6 | | | | 11089039 | | | **ELOVL2** | |  |
| rs9295757 | | | t | | 0.0436 | | | 8.58E-39 | | | | 6 | | | | 11141611 | | | **ELOVL2** | |  |
| rs3798723 | | | a | | 0.0436 | | | 8.63E-39 | | | | 6 | | | | 11149706 | | | **ELOVL2** | |  |
| rs2180725 | | | t | | -0.0436 | | | 9.58E-39 | | | | 6 | | | | 11133406 | | | **ELOVL2** | |  |
| rs1321535 | | | t | | -0.0423 | | | 1.10E-38 | | | | 6 | | | | 11184012 | | | HERV-FRD | |  |
| rs2281591 | | | a | | -0.0437 | | | 1.58E-38 | | | | 6 | | | | 11098479 | | | **ELOVL2** | |  |
| rs2295601 | | | a | | 0.0435 | | | 1.68E-38 | | | | 6 | | | | 11113672 | | | **ELOVL2** | |  |
| rs4713165 | | | t | | -0.0421 | | | 2.00E-38 | | | | 6 | | | | 11182288 | | | HERV-FRD | |  |
| rs3798719 | | | t | | 0.043 | | | 2.29E-38 | | | | 6 | | | | 11144811 | | | **ELOVL2** | |  |
| rs12665478 | | | a | | 0.0421 | | | 2.39E-38 | | | | 6 | | | | 11188811 | | | HERV-FRD | |  |
| rs12526913 | | | a | | -0.0422 | | | 2.63E-38 | | | | 6 | | | | 11190910 | | | HERV-FRD | |  |
| rs4713169 | | | c | | 0.0423 | | | 2.82E-38 | | | | 6 | | | | 11192540 | | | HERV-FRD | |  |
| rs7744440 | | | t | | -0.0429 | | | 5.26E-38 | | | | 6 | | | | 11146497 | | | **ELOVL2** | |  |
| rs3756963 | | | t | | -0.0434 | | | 5.61E-38 | | | | 6 | | | | 11130140 | | | **ELOVL2** | |  |
| rs4711171 | | | t | | 0.0414 | | | 2.45E-37 | | | | 6 | | | | 11182333 | | | HERV-FRD | |  |
| rs509360 | | | a | | 0.0456 | | | 1.04E-36 | | | | 11 | | | | 61305135 | | | **C11orf9** | |  |
| rs4713103 | | | t | | 0.0389 | | | 2.77E-36 | | | | 6 | | | | 11077127 | | | **SYCP2L** | |  |
| rs3798722 | | | a | | -0.0424 | | | 2.98E-36 | | | | 6 | | | | 11148409 | | | **ELOVL2** | |  |
| rs9295733 | | | a | | 0.0387 | | | 6.65E-36 | | | | 6 | | | | 11073433 | | | **SYCP2L** | |  |
| rs2295600 | | | a | | -0.0387 | | | 7.51E-36 | | | | 6 | | | | 11070236 | | | **SYCP2L** | |  |
| rs7774711 | | | a | | -0.0386 | | | 9.08E-36 | | | | 6 | | | | 11073007 | | | **SYCP2L** | |  |
| rs6920247 | | | c | | -0.0389 | | | 1.13E-35 | | | | 6 | | | | 11068159 | | | **SYCP2L** | |  |
| rs6918936 | | | c | | 0.0389 | | | 1.21E-35 | | | | 6 | | | | 11068144 | | | **SYCP2L** | |  |
| rs1225717 | | | a | | -0.0384 | | | 1.25E-35 | | | | 6 | | | | 11086226 | | | ELOVL2 | |  |
| rs4711146 | | | t | | -0.0388 | | | 1.32E-35 | | | | 6 | | | | 11069391 | | | **SYCP2L** | |  |
| rs3798721 | | | a | | -0.041 | | | 2.47E-35 | | | | 6 | | | | 11148169 | | | **ELOVL2** | |  |
| rs174468 | | | a | | 0.0424 | | | 3.26E-35 | | | | 11 | | | | 61420267 | | | RAB3IL1 | |  |
| rs174476 | | | t | | 0.0408 | | | 3.35E-33 | | | | 11 | | | | 61430694 | | | **RAB3IL1** | |  |
| rs666870 | | | a | | 0.0408 | | | 3.40E-33 | | | | 11 | | | | 61434055 | | | **RAB3IL1** | |  |
| rs174478 | | | t | | -0.0406 | | | 4.06E-33 | | | | 11 | | | | 61435152 | | | **RAB3IL1** | |  |
| rs1323739 | | | c | | -0.0335 | | | 1.41E-31 | | | | 6 | | | | 11112547 | | | **ELOVL2** | |  |
| rs174479 | | | c | | 0.0545 | | | 3.04E-31 | | | | 11 | | | | 61435330 | | | **RAB3IL1** | |  |
| rs2327325 | | | a | | 0.0435 | | | 4.50E-31 | | | | 6 | | | | 11073640 | | | **SYCP2L** | |  |
| rs17674802 | | | a | | 0.0418 | | | 4.78E-30 | | | | 6 | | | | 11077505 | | | **SYCP2L** | |  |
| rs9348766 | | | t | | 0.0418 | | | 5.55E-30 | | | | 6 | | | | 11076568 | | | **SYCP2L** | |  |
| rs174627 | | | a | | -0.0444 | | | 6.07E-30 | | | | 11 | | | | 61394042 | | | FADS2 | |  |
| rs9791208 | | | t | | -0.0419 | | | 7.94E-30 | | | | 6 | | | | 11069635 | | | **SYCP2L** | |  |
| rs9368506 | | | t | | 0.0416 | | | 3.12E-29 | | | | 6 | | | | 11066458 | | | **SYCP2L** | |  |
| rs412334 | | | t | | 0.0554 | | | 1.40E-26 | | | | 11 | | | | 61316837 | | | **FEN1** | |  |
| rs174469 | | | t | | -0.0717 | | | 8.14E-26 | | | | 11 | | | | 61424019 | | | **RAB3IL1** | |  |
| rs7773173 | | | c | | 0.0287 | | | 6.21E-24 | | | | 6 | | | | 11064689 | | | **SYCP2L** | |  |
| rs1225753 | | | a | | -0.0283 | | | 1.99E-23 | | | | 6 | | | | 11064675 | | | **SYCP2L** | |  |
| rs1225744 | | | t | | -0.0278 | | | 3.05E-23 | | | | 6 | | | | 11062526 | | | **SYCP2L** | |  |
| rs1225741 | | | a | | -0.0281 | | | 6.27E-23 | | | | 6 | | | | 11060229 | | | **SYCP2L** | |  |
| rs1007323 | | | t | | 0.0281 | | | 7.68E-23 | | | | 6 | | | | 11061230 | | | **SYCP2L** | |  |
| rs740006 | | | t | | -0.0764 | | | 4.50E-22 | | | | 11 | | | | 61314444 | | | **C11orf10** | |  |
| rs7394871 | | | a | | -0.0637 | | | 3.56E-16 | | | | 11 | | | | 61409090 | | | **FADS3** | |  |
| rs12207488 | | | a | | 0.0274 | | | 4.87E-16 | | | | 6 | | | | 11060322 | | | **SYCP2L** | |  |
| rs9379969 | | | a | | 0.0274 | | | 5.39E-16 | | | | 6 | | | | 11060622 | | | **SYCP2L** | |  |
| rs498793 | | | t | | 0.0307 | | | 5.84E-16 | | | | 11 | | | | 61381281 | | | **FADS2** | |  |
| rs12199131 | | | a | | 0.0267 | | | 7.69E-16 | | | | 6 | | | | 11040555 | | | **SYCP2L** | |  |
| rs12214825 | | | t | | 0.0274 | | | 1.05E-15 | | | | 6 | | | | 11041364 | | | **SYCP2L** | |  |
| rs650436 | | | t | | 0.0254 | | | 1.11E-15 | | | | 11 | | | | 61293006 | | | **C11orf9** | |  |
| rs17156426 | | | a | | 0.0551 | | | 5.02E-15 | | | | 11 | | | | 61365899 | | | **FADS2** | |  |
| rs9393800 | | | a | | -0.0266 | | | 6.10E-15 | | | | 6 | | | | 11059723 | | | **SYCP2L** | |  |
| rs7942717 | | | a | | 0.0677 | | | 8.49E-15 | | | | 11 | | | | 61403864 | | | **FADS3** | |  |
| rs579383 | | | a | | -0.0241 | | | 1.12E-14 | | | | 11 | | | | 61293159 | | | **C11orf9** | |  |
| rs17764935 | | | a | | -0.0583 | | | 1.17E-14 | | | | 11 | | | | 61421333 | | | RAB3IL1 | |  |
| rs9461310 | | | a | | -0.025 | | | 4.13E-14 | | | | 6 | | | | 11028182 | | | **SYCP2L** | |  |
| rs174472 | | | a | | 0.0274 | | | 5.74E-14 | | | | 11 | | | | 61428532 | | | **RAB3IL1** | |  |
| rs12213249 | | | t | | 0.0255 | | | 7.55E-14 | | | | 6 | | | | 11034928 | | | **SYCP2L** | |  |
| rs9366669 | | | a | | 0.0249 | | | 1.10E-13 | | | | 6 | | | | 11016855 | | | **SYCP2L** | |  |
| rs12200867 | | | c | | 0.0255 | | | 1.30E-13 | | | | 6 | | | | 11047810 | | | **SYCP2L** | |  |
| rs17762402 | | | a | | 0.0805 | | | 1.42E-13 | | | | 11 | | | | 61309777 | | | **C11orf9** | |  |
| rs2327323 | | | c | | 0.0252 | | | 2.26E-13 | | | | 6 | | | | 11041799 | | | **SYCP2L** | |  |
| rs1578068 | | | t | | 0.0251 | | | 2.32E-13 | | | | 6 | | | | 11046090 | | | **SYCP2L** | |  |
| rs9393804 | | | t | | -0.0225 | | | 2.33E-13 | | | | 6 | | | | 11064116 | | | **SYCP2L** | |  |
| rs2235093 | | | a | | 0.0263 | | | 5.36E-13 | | | | 11 | | | | 61421698 | | | **RAB3IL1** | |  |
| rs6928281 | | | t | | 0.0226 | | | 8.04E-13 | | | | 6 | | | | 11016903 | | | **SYCP2L** | |  |
| rs4713044 | | | a | | 0.0225 | | | 9.07E-13 | | | | 6 | | | | 11019268 | | | **SYCP2L** | |  |
| rs13966 | | | t | | 0.0248 | | | 9.92E-13 | | | | 11 | | | | 61421568 | | | **RAB3IL1** | |  |
| rs7759825 | | | t | | -0.0218 | | | 1.18E-12 | | | | 6 | | | | 11064635 | | | **SYCP2L** | |  |
| rs12190237 | | | a | | -0.0228 | | | 1.58E-12 | | | | 6 | | | | 11030624 | | | **SYCP2L** | |  |
| rs198464 | | | a | | 0.0191 | | | 7.47E-12 | | | | 11 | | | | 61278197 | | | C11orf9 | |  |
| rs198462 | | | a | | 0.0193 | | | 7.64E-12 | | | | 11 | | | | 61280695 | | | **C11orf9** | |  |
| rs198476 | | | a | | 0.0193 | | | 8.38E-12 | | | | 11 | | | | 61282306 | | | **C11orf9** | |  |
| rs17156442 | | | t | | -0.0513 | | | 2.09E-11 | | | | 11 | | | | 61370599 | | | **FADS2** | |  |
| rs7935946 | | | t | | -0.0482 | | | 4.72E-11 | | | | 11 | | | | 61372118 | | | **FADS2** | |  |
| rs9467921 | | | c | | -0.0211 | | | 1.43E-10 | | | | 6 | | | | 11018605 | | | **SYCP2L** | |  |
| rs17675073 | | | a | | -0.027 | | | 3.54E-10 | | | | 6 | | | | 11116635 | | | **ELOVL2** | |  |
| rs9366664 | | | t | | 0.0175 | | | 4.18E-10 | | | | 6 | | | | 11000485 | | | **SYCP2L** | |  |
| rs569258 | | | t | | -0.0187 | | | 4.37E-10 | | | | 11 | | | | 61277244 | | | C11orf9 | |  |
| rs946350 | | | c | | 0.0175 | | | 4.39E-10 | | | | 6 | | | | 11000939 | | | **SYCP2L** | |  |
| rs12195587 | | | a | | -0.0269 | | | 4.51E-10 | | | | 6 | | | | 11097928 | | | **ELOVL2** | |  |
| rs17764324 | | | t | | -0.0284 | | | 1.05E-09 | | | | 11 | | | | 61391664 | | | FADS2 | |  |
| rs17831757 | | | t | | 0.0283 | | | 1.16E-09 | | | | 11 | | | | 61391776 | | | FADS2 | |  |
| rs916924 | | | t | | 0.0469 | | | 1.19E-09 | | | | 11 | | | | 61375757 | | | **FADS2** | |  |
| rs11230815 | | | c | | 0.0283 | | | 1.22E-09 | | | | 11 | | | | 61392702 | | | FADS2 | |  |
| rs3846851 | | | a | | 0.0205 | | | 1.24E-09 | | | | 6 | | | | 11144570 | | | **ELOVL2** | |  |
| rs7104849 | | | a | | 0.0281 | | | 1.42E-09 | | | | 11 | | | | 61394620 | | | FADS3 | |  |
| rs2521572 | | | t | | -0.0497 | | | 2.17E-09 | | | | 11 | | | | 61468051 | | | BEST1 | |  |
| rs7482316 | | | a | | 0.0275 | | | 2.63E-09 | | | | 11 | | | | 61396774 | | | FADS3 | |  |
| rs198426 | | | t | | 0.0179 | | | 3.22E-09 | | | | 11 | | | | 61247062 | | | **DAGLA** | |  |
| rs3734397 | | | a | | 0.0191 | | | 4.48E-09 | | | | 6 | | | | 11090834 | | | **ELOVL2** | |  |
| rs1109748 | | | a | | -0.04 | | | 5.09E-09 | | | | 11 | | | | 61479221 | | | **BEST1** | |  |
| rs976081 | | | t | | 0.0187 | | | 6.20E-09 | | | | 6 | | | | 11111861 | | | **ELOVL2** | |  |
| rs780094 | | | t | | 0.0167 | | | 9.04E-09 | | | | 2 | | | | 27594741 | | | **GCKR** | |  |
| rs6936315 | | | t | | 0.0244 | | | 1.34E-08 | | | | 6 | | | | 11143958 | | | **ELOVL2** | |  |
| rs1260326 | | | t | | 0.0165 | | | 1.44E-08 | | | | 2 | | | | 27584444 | | | **GCKR** | |  |
| rs695867 | | | a | | 0.04 | | | 1.52E-08 | | | | 11 | | | | 61317864 | | | **FEN1** | |  |
| rs1692120 | | | a | | 0.017 | | | 1.54E-08 | | | | 11 | | | | 61174048 | | | DAGLA | |  |
| rs6456745 | | | a | | 0.0166 | | | 1.54E-08 | | | | 6 | | | | 11002254 | | | **SYCP2L** | |  |
| rs780093 | | | t | | 0.0163 | | | 2.04E-08 | | | | 2 | | | | 27596107 | | | **GCKR** | |  |
| rs1359159 | | | c | | -0.0237 | | | 3.30E-08 | | | | 6 | | | | 11042357 | | | **SYCP2L** | |  |
| rs9368453 | | | a | | -0.0157 | | | 5.90E-08 | | | | 6 | | | | 11002241 | | | **SYCP2L** | |  |
| rs9368452 | | | t | | -0.0157 | | | 6.09E-08 | | | | 6 | | | | 11001665 | | | **SYCP2L** | |  |
| rs9368446 | | | a | | -0.0156 | | | 6.56E-08 | | | | 6 | | | | 11000206 | | | **SYCP2L** | |  |
| rs10792320 | | | a | | 0.016 | | | 8.49E-08 | | | | 11 | | | | 61502867 | | | FTH1 | |  |
| rs2727261 | | | t | | -0.0368 | | | 8.85E-08 | | | | 11 | | | | 61468707 | | | BEST1 | |  |
| rs2727266 | | | a | | 0.0308 | | | 8.86E-08 | | | | 11 | | | | 61460910 | | | BEST1 | |  |
| rs1800009 | | | t | | 0.016 | | | 1.06E-07 | | | | 11 | | | | 61486810 | | | **BEST1** | |  |
| rs2521568 | | | c | | -0.0306 | | | 1.10E-07 | | | | 11 | | | | 61457509 | | | RAB3IL1 | |  |
| rs3758977 | | | t | | 0.0159 | | | 1.14E-07 | | | | 11 | | | | 61493820 | | | FTH1 | |  |
| rs2028062 | | | a | | -0.0157 | | | 1.35E-07 | | | | 11 | | | | 61502529 | | | FTH1 | |  |
| rs7435 | | | a | | -0.0173 | | | 2.40E-07 | | | | 21 | | | | 44228766 | | | **AGPAT3** | |  |
| rs1058694 | | | t | | 0.0171 | | | 3.29E-07 | | | | 21 | | | | 44228548 | | | **AGPAT3** | |  |
| rs9586179 | | | t | | 0.052 | | | 3.81E-07 | | | | 13 | | | | 102858088 | | | SLC10A2 | |  |
| rs9586178 | | | t | | -0.0516 | | | 4.19E-07 | | | | 13 | | | | 102858019 | | | SLC10A2 | |  |
| rs9586180 | | | t | | 0.0516 | | | 4.19E-07 | | | | 13 | | | | 102858134 | | | SLC10A2 | |  |
| rs1330767 | | | t | | -0.0517 | | | 4.21E-07 | | | | 13 | | | | 102858592 | | | SLC10A2 | |  |
| rs9586177 | | | t | | -0.0517 | | | 4.21E-07 | | | | 13 | | | | 102857931 | | | SLC10A2 | |  |
| rs7490068 | | | a | | -0.0516 | | | 4.26E-07 | | | | 13 | | | | 102858396 | | | SLC10A2 | |  |
| rs198428 | | | a | | 0.0152 | | | 4.62E-07 | | | | 11 | | | | 61246281 | | | **DAGLA** | |  |
| rs3117181 | | | c | | 0.0234 | | | 4.66E-07 | | | | 6 | | | | 32178995 | | | **TNXB** | |  |
| rs12587311 | | | t | | -0.0142 | | | 5.31E-07 | | | | 14 | | | | 28186446 | | | FOXG1 | |  |
| rs1225736 | | | a | | 0.016 | | | 5.33E-07 | | | | 6 | | | | 11047375 | | | **SYCP2L** | |  |
| rs4963452 | | | t | | 0.0235 | | | 5.63E-07 | | | | 11 | | | | 61572379 | | | INCENP | |  |
| rs6844153 | | | t | | 0.0204 | | | 5.94E-07 | | | | 4 | | | | 26553412 | | | **STIM2** | |  |
| rs9357021 | | | a | | -0.0168 | | | 6.18E-07 | | | | 6 | | | | 11014140 | | | **SYCP2L** | |  |
| rs8012543 | | | a | | 0.0153 | | | 6.60E-07 | | | | 14 | | | | 28157301 | | | FOXG1 | |  |
| rs10220265 | | | a | | -0.0536 | | | 6.67E-07 | | | | 13 | | | | 102847584 | | | SLC10A2 | |  |
| rs2453710 | | | a | | -0.0154 | | | 7.50E-07 | | | | 11 | | | | 61163118 | | | DAGLA | |  |
| rs2911711 | | | a | | -0.0144 | | | 7.65E-07 | | | | 2 | | | | 27604050 | | | GCKR | |  |
| rs1260333 | | | a | | 0.0144 | | | 7.67E-07 | | | | 2 | | | | 27602128 | | | GCKR | |  |
| rs11730235 | | | t | | 0.0167 | | | 8.60E-07 | | | | 4 | | | | 26533542 | | | **STIM2** | |  |
| rs2210152 | | | t | | 0.05 | | | 8.95E-07 | | | | 13 | | | | 102859587 | | | SLC10A2 | |  |
| rs2210153 | | | a | | -0.05 | | | 9.00E-07 | | | | 13 | | | | 102859642 | | | SLC10A2 | |  |
| rs3845427 | | | a | | 0.0137 | | | 9.51E-07 | | | | 1 | | | | 179529061 | | | CACNA1E | |  |
| rs3134954 | | | t | | -0.0207 | | | 1.11E-06 | | | | 6 | | | | 32179871 | | | **TNXB** | |  |
| rs2727269 | | | a | | -0.0154 | | | 1.29E-06 | | | | 11 | | | | 61467398 | | | BEST1 | |  |
| rs2838458 | | | t | | 0.0149 | | | 1.33E-06 | | | | 21 | | | | 44223084 | | | **AGPAT3** | |  |
| rs1956384 | | | t | | -0.0135 | | | 1.40E-06 | | | | 14 | | | | 28209321 | | | FOXG1 | |  |
| rs3131283 | | | t | | 0.0209 | | | 1.41E-06 | | | | 6 | | | | 32227876 | | | PRRT1 | |  |
| rs7143014 | | | a | | 0.0138 | | | 1.43E-06 | | | | 14 | | | | 28170190 | | | FOXG1 | |  |
| rs12895516 | | | c | | -0.0135 | | | 1.46E-06 | | | | 14 | | | | 28206701 | | | FOXG1 | |  |
| rs3130283 | | | a | | 0.0204 | | | 1.46E-06 | | | | 6 | | | | 32246523 | | | **AGPAT1** | |  |
| rs8004078 | | | a | | 0.014 | | | 1.46E-06 | | | | 14 | | | | 28158347 | | | FOXG1 | |  |
| rs4442733 | | | t | | 0.0135 | | | 1.50E-06 | | | | 14 | | | | 28211992 | | | FOXG1 | |  |
| rs11230767 | | | a | | 0.0146 | | | 1.59E-06 | | | | 11 | | | | 61183508 | | | DAGLA | |  |
| rs2332203 | | | a | | 0.0134 | | | 1.61E-06 | | | | 1 | | | | 179533043 | | | CACNA1E | |  |
| rs3130342 | | | a | | 0.0203 | | | 1.61E-06 | | | | 6 | | | | 32188124 | | | CREBL1 | |  |
| rs6658112 | | | c | | -0.0135 | | | 1.82E-06 | | | | 1 | | | | 179546729 | | | CACNA1E | |  |
| rs1956388 | | | a | | -0.0133 | | | 1.82E-06 | | | | 14 | | | | 28202628 | | | FOXG1 | |  |
| rs1767777 | | | a | | 0.0157 | | | 1.87E-06 | | | | 6 | | | | 11033789 | | | **SYCP2L** | |  |
| rs3129860 | | | a | | 0.0205 | | | 2.06E-06 | | | | 6 | | | | 32509057 | | | HLA-DRA | |  |
| rs3894266 | | | c | | -0.0133 | | | 2.25E-06 | | | | 1 | | | | 179556560 | | | CACNA1E | |  |
| rs10029307 | | | a | | 0.0162 | | | 2.27E-06 | | | | 4 | | | | 26513311 | | | **STIM2** | |  |
| rs3134603 | | | a | | 0.0213 | | | 2.38E-06 | | | | 6 | | | | 32233980 | | | **PPT2** | |  |
| rs3129889 | | | a | | -0.02 | | | 2.41E-06 | | | | 6 | | | | 32521523 | | | HLA-DRA | |  |
| rs6448488 | | | t | | 0.0179 | | | 2.43E-06 | | | | 4 | | | | 26560085 | | | **STIM2** | |  |
| rs3135388 | | | a | | 0.0199 | | | 2.51E-06 | | | | 6 | | | | 32521029 | | | HLA-DRA | |  |
| rs1225763 | | | a | | -0.0156 | | | 2.53E-06 | | | | 6 | | | | 11032229 | | | **SYCP2L** | |  |
| rs3792685 | | | t | | -0.0179 | | | 2.57E-06 | | | | 4 | | | | 26568305 | | | **STIM2** | |  |
| rs9268205 | | | a | | 0.0195 | | | 2.71E-06 | | | | 6 | | | | 32387916 | | | **C6orf10** | |  |
| rs3129938 | | | a | | -0.0187 | | | 2.98E-06 | | | | 6 | | | | 32444473 | | | **C6orf10** | |  |
| rs3129934 | | | t | | 0.0187 | | | 3.01E-06 | | | | 6 | | | | 32444165 | | | **C6orf10** | |  |
| rs3129868 | | | a | | 0.0207 | | | 3.06E-06 | | | | 6 | | | | 32512355 | | | HLA-DRA | |  |
| rs1800684 | | | a | | 0.021 | | | 3.14E-06 | | | | 6 | | | | 32259972 | | | **AGER** | |  |
| rs3117182 | | | a | | -0.0216 | | | 3.14E-06 | | | | 6 | | | | 32174797 | | | **TNXB** | |  |
| rs3117117 | | | a | | -0.0196 | | | 3.18E-06 | | | | 6 | | | | 32429250 | | | **C6orf10** | |  |
| rs1225732 | | | a | | 0.0156 | | | 3.24E-06 | | | | 6 | | | | 11042556 | | | **SYCP2L** | |  |
| rs1225731 | | | t | | -0.0154 | | | 3.40E-06 | | | | 6 | | | | 11040479 | | | **SYCP2L** | |  |
| rs12234119 | | | a | | 0.0142 | | | 3.50E-06 | | | | 6 | | | | 11010318 | | | **SYCP2L** | |  |
| rs3911590 | | | a | | -0.0129 | | | 3.52E-06 | | | | 1 | | | | 179528781 | | | CACNA1E | |  |
| rs9268154 | | | a | | -0.0186 | | | 3.54E-06 | | | | 6 | | | | 32373999 | | | **C6orf10** | |  |
| rs10910917 | | | a | | -0.013 | | | 3.54E-06 | | | | 1 | | | | 179531868 | | | CACNA1E | |  |
| rs3135391 | | | a | | 0.0196 | | | 3.60E-06 | | | | 6 | | | | 32518965 | | | **HLA-DRA** | |  |
| rs951500 | | | t | | -0.0389 | | | 3.65E-06 | | | | 4 | | | | 83878052 | | | **SCD5** | |  |
| rs9268148 | | | a | | -0.0185 | | | 3.66E-06 | | | | 6 | | | | 32367505 | | | C6orf10 | |  |
| rs3134943 | | | t | | 0.0196 | | | 3.70E-06 | | | | 6 | | | | 32255739 | | | **RNF5** | |  |
| rs4048562 | | | a | | 0.0129 | | | 3.76E-06 | | | | 1 | | | | 179528335 | | | CACNA1E | |  |
| rs10220061 | | | a | | 0.0506 | | | 3.85E-06 | | | | 13 | | | | 102845062 | | | SLC10A2 | |  |
| rs12649969 | | | a | | 0.0187 | | | 3.85E-06 | | | | 4 | | | | 26655420 | | | STIM2 | |  |
| rs7523745 | | | t | | -0.0129 | | | 3.90E-06 | | | | 1 | | | | 179541038 | | | CACNA1E | |  |
| rs3129900 | | | t | | -0.0186 | | | 3.90E-06 | | | | 6 | | | | 32413957 | | | **C6orf10** | |  |
| rs7654400 | | | a | | 0.0188 | | | 4.09E-06 | | | | 4 | | | | 26657640 | | | STIM2 | |  |
| rs2332206 | | | a | | -0.0129 | | | 4.10E-06 | | | | 1 | | | | 179540465 | | | CACNA1E | |  |
| rs2642438 | | | a | | -0.0154 | | | 4.20E-06 | | | | 1 | | | | 219036651 | | | **MOSC1** | |  |
| rs9313625 | | | a | | -0.0146 | | | 4.20E-06 | | | | 5 | | | | 172622830 | | | NKX2-5 | |  |
| rs3104391 | | | t | | 0.0212 | | | 4.40E-06 | | | | 6 | | | | 32702879 | | | HLA-DQA1 | |  |
| rs4706778 | | | a | | -0.0195 | | | 4.50E-06 | | | | 6 | | | | 80121944 | | | HMGN3 | |  |
| rs6457535 | | | a | | 0.0194 | | | 4.50E-06 | | | | 6 | | | | 32370384 | | | **C6orf10** | |  |
| rs3117189 | | | a | | 0.0245 | | | 4.63E-06 | | | | 6 | | | | 32141922 | | | **TNXB** | |  |
| **D. Comprehensive results for DHA with p <5*10^-6^** | | | | | | | | | | | | | | | | | | | | | |
| MarkerName | | | Effect allele | | | | Effect* | | | P.value | | | | Chr | | Position | | | Nearest Gene** | | |
| rs2236212 | | | C | | | | -0.1132 | | | 1.26E-15 | | | | 6 | | 11103001 | | | **ELOVL2** | | |
| rs3798713 | | | c | | | | -0.1126 | | | 1.40E-15 | | | | 6 | | 11116608 | | | **ELOVL2** | | |
| rs3734398 | | | t | | | | 0.1143 | | | 1.65E-15 | | | | 6 | | 11090959 | | | **ELOVL2** | | |
| rs953413 | | | a | | | | -0.1108 | | | 2.98E-15 | | | | 6 | | 11120845 | | | **ELOVL2** | | |
| rs4532436 | | | c | | | | 0.1128 | | | 3.17E-15 | | | | 6 | | 11091957 | | | **ELOVL2** | | |
| rs3798707 | | | t | | | | -0.1112 | | | 3.27E-15 | | | | 6 | | 11099921 | | | **ELOVL2** | | |
| rs2295602 | | | t | | | | 0.1108 | | | 3.30E-15 | | | | 6 | | 11113828 | | | **ELOVL2** | | |
| rs3798711 | | | t | | | | 0.1107 | | | 3.54E-15 | | | | 6 | | 11110796 | | | **ELOVL2** | | |
| rs1570069 | | | a | | | | 0.1105 | | | 3.61E-15 | | | | 6 | | 11125811 | | | **ELOVL2** | | |
| rs1225737 | | | t | | | | -0.1125 | | | 3.63E-15 | | | | 6 | | 11090638 | | | **ELOVL2** | | |
| rs7743830 | | | a | | | | 0.1105 | | | 3.65E-15 | | | | 6 | | 11122206 | | | **ELOVL2** | | |
| rs1321536 | | | t | | | | 0.1106 | | | 6.04E-15 | | | | 6 | | 11126798 | | | **ELOVL2** | | |
| rs9295763 | | | c | | | | -0.1165 | | | 9.87E-15 | | | | 6 | | 11153178 | | | ELOVL2 | | |
| rs9295764 | | | a | | | | 0.1166 | | | 1.00E-14 | | | | 6 | | 11153182 | | | ELOVL2 | | |
| rs1323739 | | | c | | | | 0.108 | | | 2.00E-14 | | | | 6 | | 11112547 | | | **ELOVL2** | | |
| rs17675322 | | | a | | | | -0.1169 | | | 3.50E-14 | | | | 6 | | 11167171 | | | ELOVL2 | | |
| rs8523 | | | a | | | | -0.1141 | | | 3.94E-14 | | | | 6 | | 11089039 | | | **ELOVL2** | | |
| rs6900220 | | | t | | | | 0.1173 | | | 4.50E-14 | | | | 6 | | 11173124 | | | ELOVL2 | | |
| rs2147041 | | | a | | | | 0.1169 | | | 5.02E-14 | | | | 6 | | 11158509 | | | ELOVL2 | | |
| rs1225717 | | | a | | | | 0.113 | | | 7.13E-14 | | | | 6 | | 11086226 | | | ELOVL2 | | |
| rs4713103 | | | t | | | | -0.1129 | | | 7.83E-14 | | | | 6 | | 11077127 | | | **SYCP2L** | | |
| rs7774711 | | | a | | | | 0.1122 | | | 1.25E-13 | | | | 6 | | 11073007 | | | **SYCP2L** | | |
| rs9295733 | | | a | | | | -0.1122 | | | 1.34E-13 | | | | 6 | | 11073433 | | | **SYCP2L** | | |
| rs2295600 | | | a | | | | 0.1114 | | | 1.99E-13 | | | | 6 | | 11070236 | | | **SYCP2L** | | |
| rs4711146 | | | t | | | | 0.1114 | | | 2.84E-13 | | | | 6 | | 11069391 | | | **SYCP2L** | | |
| rs6920247 | | | c | | | | 0.1114 | | | 2.92E-13 | | | | 6 | | 11068159 | | | **SYCP2L** | | |
| rs6918936 | | | c | | | | -0.1114 | | | 2.99E-13 | | | | 6 | | 11068144 | | | **SYCP2L** | | |
| rs4711171 | | | t | | | | -0.1158 | | | 4.61E-13 | | | | 6 | | 11182333 | | | HERV-FRD | | |
| rs1321535 | | | t | | | | 0.1151 | | | 7.12E-13 | | | | 6 | | 11184012 | | | HERV-FRD | | |
| rs12665478 | | | a | | | | -0.1152 | | | 7.12E-13 | | | | 6 | | 11188811 | | | HERV-FRD | | |
| rs4713165 | | | t | | | | 0.115 | | | 7.23E-13 | | | | 6 | | 11182288 | | | HERV-FRD | | |
| rs12526913 | | | a | | | | 0.1155 | | | 7.36E-13 | | | | 6 | | 11190910 | | | HERV-FRD | | |
| rs4713169 | | | c | | | | -0.1158 | | | 7.48E-13 | | | | 6 | | 11192540 | | | HERV-FRD | | |
| rs3798721 | | | a | | | | 0.1127 | | | 2.32E-12 | | | | 6 | | 11148169 | | | **ELOVL2** | | |
| rs3798723 | | | a | | | | -0.1129 | | | 4.39E-12 | | | | 6 | | 11149706 | | | **ELOVL2** | | |
| rs9393903 | | | a | | | | -0.1127 | | | 7.29E-12 | | | | 6 | | 11150895 | | | **ELOVL2** | | |
| rs3798719 | | | t | | | | -0.1111 | | | 8.42E-12 | | | | 6 | | 11144811 | | | **ELOVL2** | | |
| rs3798722 | | | a | | | | 0.1113 | | | 1.20E-11 | | | | 6 | | 11148409 | | | **ELOVL2** | | |
| rs9295757 | | | t | | | | -0.1105 | | | 1.65E-11 | | | | 6 | | 11141611 | | | **ELOVL2** | | |
| rs1007323 | | | t | | | | -0.0947 | | | 2.20E-11 | | | | 6 | | 11061230 | | | **SYCP2L** | | |
| rs2180725 | | | t | | | | 0.1096 | | | 2.33E-11 | | | | 6 | | 11133406 | | | **ELOVL2** | | |
| rs1225741 | | | a | | | | 0.0946 | | | 2.35E-11 | | | | 6 | | 11060229 | | | **SYCP2L** | | |
| rs7744440 | | | t | | | | 0.1081 | | | 2.58E-11 | | | | 6 | | 11146497 | | | **ELOVL2** | | |
| rs17606561 | | | a | | | | -0.1108 | | | 3.43E-11 | | | | 6 | | 11090345 | | | **ELOVL2** | | |
| rs2295601 | | | a | | | | -0.1085 | | | 3.51E-11 | | | | 6 | | 11113672 | | | **ELOVL2** | | |
| rs3756963 | | | t | | | | 0.109 | | | 4.07E-11 | | | | 6 | | 11130140 | | | **ELOVL2** | | |
| rs2281591 | | | a | | | | 0.1082 | | | 4.29E-11 | | | | 6 | | 11098479 | | | **ELOVL2** | | |
| rs7773173 | | | c | | | | -0.0925 | | | 5.42E-11 | | | | 6 | | 11064689 | | | **SYCP2L** | | |
| rs9368564 | | | a | | | | 0.1144 | | | 5.96E-11 | | | | 6 | | 11168269 | | | ELOVL2 | | |
| rs1225753 | | | a | | | | 0.0915 | | | 7.10E-11 | | | | 6 | | 11064675 | | | **SYCP2L** | | |
| rs1225744 | | | t | | | | 0.0894 | | | 1.28E-10 | | | | 6 | | 11062526 | | | **SYCP2L** | | |
| rs9393915 | | | t | | | | -0.1126 | | | 2.66E-10 | | | | 6 | | 11180308 | | | ELOVL2 | | |
| rs2327325 | | | a | | | | -0.1018 | | | 7.24E-09 | | | | 6 | | 11073640 | | | **SYCP2L** | | |
| rs9368506 | | | t | | | | -0.1011 | | | 7.38E-09 | | | | 6 | | 11066458 | | | **SYCP2L** | | |
| rs17674802 | | | a | | | | -0.0995 | | | 8.21E-09 | | | | 6 | | 11077505 | | | **SYCP2L** | | |
| rs9348766 | | | t | | | | -0.0993 | | | 9.03E-09 | | | | 6 | | 11076568 | | | **SYCP2L** | | |
| rs9791208 | | | t | | | | 0.0997 | | | 9.81E-09 | | | | 6 | | 11069635 | | | **SYCP2L** | | |
| rs12324142 | | | t | | | | 0.3053 | | | 1.03E-06 | | | | 15 | | 74201944 | | | **C15orf27** | | |
| rs3846851 | | | a | | | | -0.0821 | | | 1.05E-06 | | | | 6 | | 11144570 | | | **ELOVL2** | | |
| rs174555 | | | t | | | | 0.0758 | | | 1.09E-06 | | | | 11 | | 61336336 | | | **FADS1** | | |
| rs2935980 | | | t | | | | 0.303 | | | 1.13E-06 | | | | 15 | | 74209208 | | | **C15orf27** | | |
| rs174548 | | | c | | | | 0.0757 | | | 1.18E-06 | | | | 11 | | 61327924 | | | **FADS1** | | |
| rs174549 | | | a | | | | -0.0757 | | | 1.27E-06 | | | | 11 | | 61327958 | | | **FADS1** | | |
| rs1842717 | | | t | | | | 0.5521 | | | 1.55E-06 | | | | 8 | | 73279013 | | | TRPA1 | | |
| rs10898527 | | | a | | | | -0.2292 | | | 1.89E-06 | | | | 11 | | 86118787 | | | ME3 | | |
| rs16935203 | | | c | | | | 0.1809 | | | 2.06E-06 | | | | 11 | | 40935017 | | | LRRC4C | | |
| rs16935201 | | | a | | | | -0.1809 | | | 2.06E-06 | | | | 11 | | 40934167 | | | LRRC4C | | |
| rs102275 | | | t | | | | 0.0702 | | | 2.19E-06 | | | | 11 | | 61314379 | | | **C11orf10** | | |
| rs976081 | | | t | | | | -0.0759 | | | 2.39E-06 | | | | 6 | | 11111861 | | | **ELOVL2** | | |
| rs2236709 | | | a | | | | -0.0748 | | | 2.67E-06 | | | | 11 | | 112691986 | | | **TTC12** | | |
| rs16935194 | | | t | | | | 0.1801 | | | 2.74E-06 | | | | 11 | | 40933178 | | | LRRC4C | | |
| rs174550 | | | t | | | | 0.0696 | | | 2.82E-06 | | | | 11 | | 61328054 | | | **FADS1** | | |
| rs16967934 | | | t | | | | 0.2642 | | | 2.91E-06 | | | | 15 | | 74243975 | | | **C15orf27** | | |
| rs3734397 | | | a | | | | -0.076 | | | 3.00E-06 | | | | 6 | | 11090834 | | | **ELOVL2** | | |
| rs7163654 | | | a | | | | -0.2456 | | | 3.17E-06 | | | | 15 | | 74243506 | | | **C15orf27** | | |
| rs174538 | | | a | | | | -0.0726 | | | 3.34E-06 | | | | 11 | | 61316657 | | | **C11orf10** | | |
| rs174546 | | | t | | | | -0.069 | | | 3.35E-06 | | | | 11 | | 61326406 | | | **FADS1** | | |
| rs174576 | | | a | | | | -0.0699 | | | 3.40E-06 | | | | 11 | | 61360086 | | | **FADS2** | | |
| rs174547 | | | t | | | | 0.0691 | | | 3.42E-06 | | | | 11 | | 61327359 | | | **FADS1** | | |
| rs174545 | | | c | | | | 0.069 | | | 3.48E-06 | | | | 11 | | 61325882 | | | **FADS1** | | |
| rs1391470 | | | t | | | | -0.2398 | | | 3.82E-06 | | | | 15 | | 74216840 | | | **C15orf27** | | |
| rs4427557 | | | t | | | | 0.2024 | | | 3.87E-06 | | | | 11 | | 40818332 | | | LRRC4C | | |
| rs174574 | | | a | | | | -0.0687 | | | 3.96E-06 | | | | 11 | | 61356918 | | | **FADS2** | | |
| rs174536 | | | a | | | | 0.0687 | | | 4.06E-06 | | | | 11 | | 61308503 | | | **C11orf9** | | |
| rs174535 | | | t | | | | 0.0686 | | | 4.14E-06 | | | | 11 | | 61307932 | | | **C11orf9** | | |
| rs174537 | | | t | | | | -0.0685 | | | 4.29E-06 | | | | 11 | | 61309256 | | | **C11orf9** | | |
| rs174601 | | | t | | | | -0.0756 | | | 4.30E-06 | | | | 11 | | 61379716 | | | **FADS2** | | |
| rs174577 | | | a | | | | -0.0691 | | | 4.40E-06 | | | | 11 | | 61361390 | | | **FADS2** | | |
| rs174578 | | | a | | | | -0.0692 | | | 4.51E-06 | | | | 11 | | 61362075 | | | **FADS2** | | |

**E. Results for DPA adjusted for rs2236212 and rs174547 with p < 5*10^-8^**

| MarkerName | Effect Allele | Effect* | P.value | Chr | Position | Nearest Gene** | |
| --- | --- | --- | --- | --- | --- | --- | --- |
| rs12662634 | a | -0.0299 | 2.68E-10 | 6 | 11182177 | ELOVL2 | |
| rs4711170 | a | -0.0271 | 1.24E-09 | 6 | 11178420 | ELOVL2 | |
| rs9368564 | a | -0.0265 | 1.41E-09 | 6 | 11168269 | ELOVL2 | |
| rs17764682 | a | 0.0263 | 2.60E-09 | 6 | 11166801 | ELOVL2 | |
| rs9393915 | t | 0.0262 | 3.38E-09 | 6 | 11180308 | ELOVL2 | |
| rs17606561 | a | 0.0247 | 5.05E-09 | 6 | 11090345 | **ELOVL2** | |
| rs9468304 | a | -0.0249 | 5.11E-09 | 6 | 11150151 | **ELOVL2** | |
| rs10498676 | a | -0.0241 | 5.79E-09 | 6 | 11134985 | **ELOVL2** | |
| rs3798710 | c | -0.0239 | 6.45E-09 | 6 | 11110769 | **ELOVL2** | |
| rs17764592 | a | 0.0239 | 6.52E-09 | 6 | 11111130 | **ELOVL2** | |
| rs2180725 | t | -0.0237 | 6.66E-09 | 6 | 11133406 | **ELOVL2** | |
| rs2281591 | a | -0.0237 | 7.82E-09 | 6 | 11098479 | **ELOVL2** | |
| rs2295601 | a | 0.0236 | 8.20E-09 | 6 | 11113672 | **ELOVL2** | |
| rs3798709 | a | 0.0237 | 8.37E-09 | 6 | 11109262 | **ELOVL2** | |
| rs9295757 | t | 0.0235 | 8.59E-09 | 6 | 11141611 | **ELOVL2** | |
| rs3778166 | a | -0.0236 | 1.16E-08 | 6 | 11141150 | **ELOVL2** | |
| rs3756963 | t | -0.0233 | 1.26E-08 | 6 | 11130140 | **ELOVL2** | |
| rs9393903 | a | 0.0233 | 1.54E-08 | 6 | 11150895 | **ELOVL2** | |
| rs3798723 | a | 0.0232 | 1.60E-08 | 6 | 11149706 | **ELOVL2** | |
| rs7744440 | t | -0.0226 | 1.77E-08 | 6 | 11146497 | **ELOVL2** | |
| rs911196 | t | 0.0231 | 2.08E-08 | 6 | 11098737 | **ELOVL2** | |
| rs3798719 | t | 0.0227 | 2.40E-08 | 6 | 11144811 | **ELOVL2** | |
| * regression coefficient associated with one copy of the effect allele | | | | | | |  |
| **Nearest reference is bolded if SNP is within the reference gene | | | | | | |  |
